# Recyclable luminescent solar concentrator from lead-free perovskite derivative

**DOI:** 10.1038/s41377-025-01973-0

**Published:** 2025-08-28

**Authors:** Huanxin Yang, Haolin Lu, Xuejiao Wang, Wenda Sun, Yujing Yang, Wei Xiong, Guankui Long, Jialiang Xu, Xiaodan Zhang, Mingjian Yuan, Xiyan Li

**Affiliations:** 1 Institute of Photoelectronic Thin Film Devices and Technology, Nankai University; Tianjin Key Laboratory of Efficient Utilization of Solar Energy, Nankai University; Engineering Research Center of Thin Film Photoelectronic Technology of Ministry of Education, Nankai University; State Key Laboratory of Photovoltaic Materials and Cells, Nankai University; Academy for Advanced Interdisciplinary Studies (AAIS), Nankai University, Tianjin, 300350 China; 2 School of Materials Science and Engineering, Nankai University, Tianjin, 300350 China; 3 Department of Opto-electronics and Information Engineering, Tianjin University; School of Precision Instruments and Opto-electronics Engineering, Tianjin University; Key Laboratory of Optoelectronic Information Technology (Ministry of Education), Tianjin University; Key Laboratory of Micro-Opto-Electro-Mechanical Systems (MOEMS) Technology (Ministry of Education), Tianjin University, Tianjin, 300072 China; 4 Shanghai Re-poly Environmental Protection Technology Co., Ltd., Shanghai, 201209 China; 5 Key Laboratory of Advanced Energy Materials Chemistry (Ministry of Education), Renewable Energy Conversion and Storage Center (RECAST), College of Chemistry, Nankai University, Tianjin, 300071 China

**Keywords:** Optical materials and structures, Applied optics

## Abstract

Luminescent solar concentrators (LSCs) offer a sustainable approach to power generation using fluorescent glasses, yet their green industrialization is impeded by the limited production scale and non-recyclability of embedded nanocrystals. Here, we introduce a lead-free perovskite derivative ETP_2_SbCl_5_ (ETP = (C_6_H_5_)_3_PC_2_H_5_) with a reversible transition between powder and glass states. Through molecular dynamics and density functional theory, we elucidate the possible structural distortions of [SbCl_5_] pyramids and their impact on luminescence. The fabricated LSCs, utilizing such fluorescent glasses with an efficient absorption for <420 nm, achieve the highest power conversion and optical efficiencies of ~5.56% and ~32.5%, respectively. In addition to self-healing by reheating at ~200 °C, impressively, it could be mass recycled to phosphor by ethanol or heating treatments, which still maintains nearly initial fluorescent performance and could be repurposed like freshly synthesized samples. This work presents a paradigm for the sustainable use of fluorescent materials and offers a reliable path toward low-carbon globalization.

## Abstract (web summary)

The ETP_2_SbCl_5_ characterized by the reversible phosphor-glass transition was developed for recyclable luminescent solar concentrators, of which the maximum power conversion and optical efficiencies reached ~5.56% and ~32.5%, respectively.

## Graphical abstract

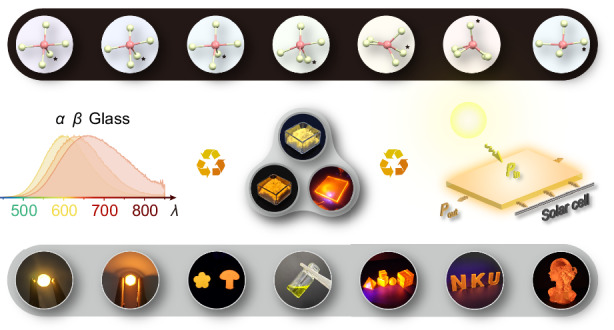

## Introduction

Luminescent solar concentrators (LSCs), which balance transparency with photovoltaic capabilities, harmoniously combining energy generation with architectural esthetics, are emerging as pivotal solutions in the quest for self-sufficient green energy^1^. Using semitransparent fluorescent glasses, LSCs absorb a portion of sunlight (such as ultraviolet light) and undergo photoluminescence (PL), which is then conducted to the solar cells integrated on the edges for optical-electrical conversion^2^. As a key component, in previous reports, the fluorescent glasses were fabricated by embedding nano-scale crystals, such as the carbon- ^3^, silicon- ^4^, and Se/S-based^5,6^ quantum dots (QDs), as well as Pb-based perovskite nanocrystals (NCs)^7–10^, into various polymers. Despite the relatively mature synthesis protocols for QDs/NCs at the laboratory stage, their protracted fabrication durations, massive solvent consumption, and constrained chemical yield result in elevated preparation costs, which present substantial challenges for future large-scale production of LSCs (Supplementary Table S1). Moreover, the embedded emitters are challenging to segregate and recycle, which leads to a disposable use characteristic, necessitating re-synthesis and re-embedding once the glasses are damaged. By comparison, one-step synthesis of fluorescent glasses by simply thermal treatment is more desirable for low-cost and mass-scale production, and exploring such new materials and corresponding preparation schemes are considered crucial to potential industrialization.

Ionic liquids, recognized for their environmental compatibility and versatile functionality, have been recently employed to modify interfaces and passivate NCs/QDs effectively^11–13^. Aided by the self-solvent property and cationic components, they could combine with metal ions thus forming (semi-)transparent glasses with zero-dimensional structures at mild heating temperatures. For example, in 2002, Mitzi et al. originally reported on a perovskite film (2-FPEA)_2_SnI_4_ (2-FPEA = C_6_H_4_FC_2_H_4_NH_3_), which possessed a relatively low melting temperature of ~200 °C, enabling its conversion into transparent thin film^14^. However, the immediate crystallization would occur once cooling to room temperature, hindering the investigation for amorphous state. Then, exploring the melting and quenching conditions for preparing stable hybrid glass became a popular research focus, of which the trend continued until that the S(1-1)NPB (*S*-(1-1)napthylethylammonium lead bromide) was demonstrated to readily enable access to a glass state through quenching from the melt in 2020 (ref. ^15,16^). Recently, with the develop of lead-free perovskites, *ns*^2^ and RE-based (RE = rare earth) hybrid luminescent glasses with shape-on-demand characteristics were gradually explored, such as (Bmmim)_2_SbCl_5_ (Bmmim = C_4_H_6_N_2_C_4_H_9_, ref. ^17^), (HTP/ETP)_2_MnBr_4_ (HTP = (C_6_H_5_)_3_PC_7_H_15_, ETP = (C_6_H_5_)_3_PC_2_H_5_, ref. ^18,19^), ETP_2_SbCl_5_ (ref. ^20,21^), and RE(NO_3_)_3_(C_5_H_2_N_4_)_2_ (ref. ^22^), etc. Although the microscopic mechanisms of the powder-glass transition and specific structural distortion are still unclear, the hybrid glasses have indeed exhibited exceptional performance in various applications including memory and computing^23,24^, energy storage^25,26^, thermoelectric^27^, photovoltaic^28^ and X-ray detection^29,30^, etc.

Such burgeoning perovskite hybrid glasses could greatly integrate the functions of fluorophores and polymers/glasses, which present an exemplary solution for the preparation strategy and chemical yields. By using their optical waveguide effect, this technique of fluorescent glass may offer a promising scheme for innovative LSCs. Additionally, in the age of low-carbon and sustainable globalization, heightened attention should be given to the recyclability of these novel fluorescent glasses once damaged or discarded, thereby eliminating disposable use, resource squandering or heavy metal pollution^31,32^.

In this work, we synthesized the yellow emissive ETP_2_SbCl_5_ phosphor with a near-unity PLQY by a simple solution process at room temperature, of which the glass phase could be obtained by further thermal treatment. The as-fabricated ETP_2_SbCl_5_-based LSCs exhibited the highest power conversion and optical efficiencies of ~5.56% and ~32.5%, respectively, on a 3 × 3 × 0.5 cm^3^ fluorescent glasses. Besides the self-healing property, reversible transitions between phosphor and glass phases have been detected. Even after undergoing 10 cycles of phosphor-glass transitions, the final recycled phosphors still maintained ~95% of their initial PL performance, enabling them to be further used in other fields, such as phosphor converted-LED or anti-counterfeiting, etc., as effective as freshly synthesized phosphors.

## Results

### Preparation and fluorescence of *α*-ETP_2_SbCl_5_

**Fig. 1:**
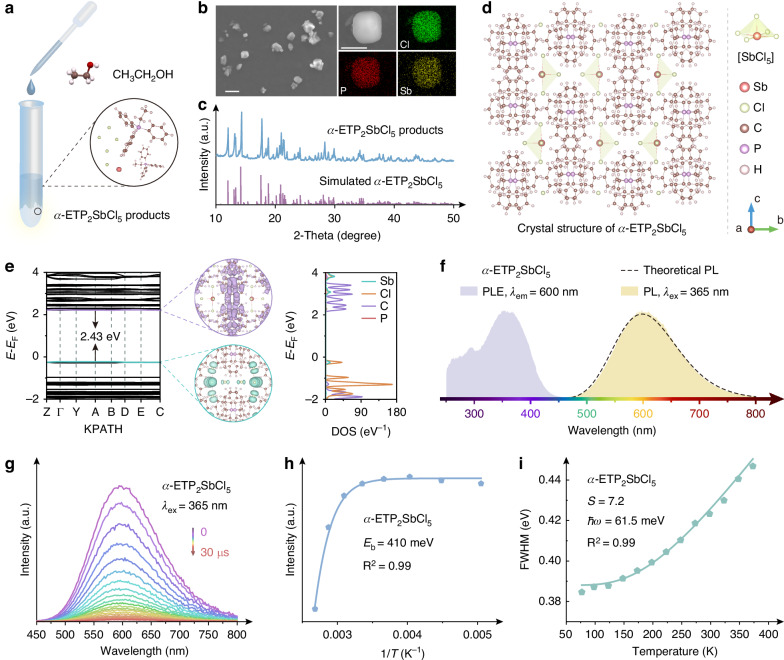
Synthesis and characterizations of *α*-ETP_2_SbCl_5_. **a** Synthesis scheme for *α*-ETP_2_SbCl_5_; **b** SEM images (scale bars = 10 μm), **c** XRD profile, and **d** Crystal structure of the *α*-ETP_2_SbCl_5_; **e** DFT calculated energy band structures and corresponding DOS profiles of *α*-ETP_2_SbCl_5_. The purple and green regions represent the distributions of CBM and VBM, respectively; **f** PLE (purple) and PL (yellow) spectra of as-synthesized *α*-ETP_2_SbCl_5_. The *λ*_em_ and *λ*_ex_ are 600 and 365 nm, respectively. The dashed line represents the theoretical PL spectrum; **g** 365 nm-excited TRPL spectra of *α*-ETP_2_SbCl_5_ within 0–30 μs; Fitting results for (**h**) *E*_b_, (**i**) *S* and *ħω* parameters

The *α*-ETP_2_SbCl_5_ (ETP = (C_6_H_5_)_3_PC_2_H_5_) powder is synthesized by simply mixing ETPCl and SbCl_3_ in moderate ethanol (Fig. 1a), with a near-100% chemical yield. Similar to our previous report, this simple solution-process strategy greatly supports rapid and mass preparation for green industrialization^33^. The as-synthesized *α*-ETP_2_SbCl_5_ exhibits a main particle size of ~1–7 μm (Fig. 1b and Supplementary Fig. S1a–c), with uniformly distributed P, Sb, and Cl elements. X-ray diffraction (XRD) patterns of the *α*-ETP_2_SbCl_5_ products are consistent with that of the standard crystal (Fig. 1c, Supplementary Fig. S2a and Table S2)^20,21^, suggesting a uniformly pure phase and the reliability of our strategy. The crystal structure of the *α*-ETP_2_SbCl_5_ is shown in Fig. 1d, where the Sb atoms bond with Cl atoms thus forming [SbCl_5_] pyramids, and they are separated by the organic units (ETP) to form perovskite derivative with a zero-dimensional structure. Figure 1e shows the energy band structure and density of state (DOS) of *α*-ETP_2_SbCl_5_ by density functional theory (DFT), in which the valence band maximum (VBM) is mainly consistent with Sb and Cl atoms, that is the [SbCl_5_] pyramids, suggesting the electrons in [SbCl_5_] pyramids could absorb the photonic energy for excitation. The conduction band minimum (CBM) is mainly from C atoms of organic units, and the formed energy band gap with VBM is ~2.43 eV. The VBM and CBM are both flat, inducing large effective masses of both holes and electrons, which lays a foundation for self-trapped excitons (STE)-based emission^34–36^.

As shown in Fig. 1f, under the photoluminescence excitation (PLE) of 365 nm, the *α*-ETP_2_SbCl_5_ products emit a bright yellow light covering from ~470 to ~800 nm (central wavelength: ~600 nm; PLQY: (97.2 ± 2.6)%), which should be assigned to the triplet STEs of Sb^3+^ ions, specifically ^3^P_1_ → ^1^S_0_ transition^37,38^, consistent with the theoretical STE emission behavior. Furthermore, photoluminescence (PL) bands at ~330 and ~450 nm could be detected under a 280 nm excitation, which could be assigned to the transitions of the band gap and the organic ETP, respectively (Supplementary Fig. S1d–f). The time-resolved PL (TRPL) profiles show consistent spectral shapes and central wavelengths (Fig. 1g and Supplementary Fig. S1g), further indicating the pure luminous center of [SbCl_5_] pyramids under 365 nm excitation, without obvious defects. With the assistance of temperature-dependent PL profiles (Supplementary Fig. S1h, i), the exciton binding energy (*E*_b_) could be fitted as ~410 meV (Fig. 1h) by Formula 1 (*T* is the testing temperature. *I*(*T*) and *I*_0_ represent the integrated emission intensities at *T* and 0 K, respectively. *A* and *k*_b_ are the constant and Boltzmann constant, respectively.), which is much higher than 26 meV, suggesting the preference of free carriers to form excitons for luminescence at room temperature^37,39,40^. Huang-Rhys factor (*S*) is commonly used for qualifying the electron-phonon coupling strength, which could be calculated by Formula 2 (ref. ^41^). The *ħω* represents phonon energy, in connection with the excited state and self-trapping time, and the *T* is temperature. The *S* value of *α*-ETP_2_SbCl_5_ is estimated as ~7.2 through fitting temperature dependence of full width at half maximum (FWHM), shown in Fig. 1i, which denotes an effective STE emission (Supplementary Fig. S3), corresponding to its PL behaviors described above^42^.

1 $$I(T)=\frac{{I}_{0}}{1+{{Ae}}^{-{E}_{b}/{k}_{b}T}}$$

2 $${FWHM}=2.36\sqrt{S}{{{\hbar }}\omega }_{{\rm{phonon}}}\sqrt{coth \frac{{{{\hbar }}\omega }_{{\rm{phonon}}}}{2{k}_{b}T}}$$

### Phase transition from *α*-, through *β*-, to G-ETP_2_SbCl_5_

**Fig. 2:**
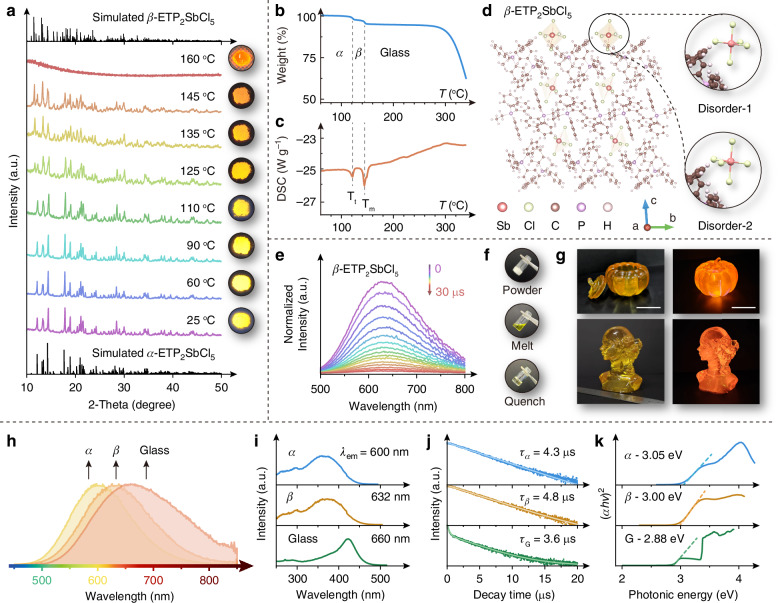
Thermal-induced phase transitions from *α*-, through *β*-, to G-ETP_2_SbCl_5_. **a** XRD profiles of as-synthesized *α*-ETP_2_SbCl_5_ products after being pre-treated by different temperatures for 10 min. The insets are the luminescent photographs of the samples after various heating pre-treatments; **b** TGA and **c** DSC profiles of the as-synthesized *α*-ETP_2_SbCl_5_; **d** Crystal structure of *β*-ETP_2_SbCl_5_. The insets are two disordered pyramids with opposite orientations; **e** TRPL spectra of *β*-ETP_2_SbCl_5_ within 0–30 μs; **f** Three states of the material before (raw powder), during (melting liquid) and after (transparent glass) heating treatment; **g** Digital photographs of the G-ETP_2_SbCl_5_ shaped like pumpkin and model girl under indoor white light (left column) and UV excitation (right column). The scale bars represent 5 cm; Comparisons in (**h**) PL, **i** PLE spectra, **j** lifetime decay curves, and **k** fitting band gaps of *α*-, *β*- and G-ETP_2_SbCl_5_

The XRD patterns in Fig. 2a show that, the *α*-ETP_2_SbCl_5_ products are relatively stable below the pre-treated temperature of ~110 °C. However, additional diffraction patterns appear while further increasing the pre-treated temperature to ~125 °C, which should be assigned to the new phase of *β*-ETP_2_SbCl_5_, and it becomes the only remaining phase after pre-heated at 135 °C for 10 min, denoting the complete phase transition from *α*- to *β*-ETP_2_SbCl_5_ (Supplementary Fig. S2b and Table S2). This phase transition could also be tracked by thermogravimetric analysis (TGA), shown as the first step in Fig. 2b (mass loss ~2%), during which the sample absorbs thermal energy and exhibits a negative differential scanning calorimetry (DSC) peak, shown as the signal at *T*_transition_ (called *T*_t_ hereafter, *T*_t_ ~ 121 °C) in Fig. 2c. In the crystal structure of the *β*-ETP_2_SbCl_5_, Sb still maintains [SbCl_5_] pyramid configuration, but with a disordered orientation. For instance, in Fig. 2d, the edge of the crystal appears two octahedrons, while each of them is actually the superposition of two possibilities, namely disorder-1 and -2 with opposite orientations, and the statistical probability that a Cl atom occupies a symmetric position is 1/2. Nevertheless, in TRPL spectra, *β*-ETP_2_SbCl_5_ still exhibits uniform orange-yellow emissions with central wavelengths of ~632 nm (Fig. 2e and Supplementary Fig. S4a, b), probably due to the similar configurations including the lengths and angles of Sb-Cl bonds between each [SbCl_5_] pyramid. The *β*-ETP_2_SbCl_5_ exhibits a similar high PLQY of (96.5 ± 3.7)% and a slightly higher *S* value of ~9.7 (Supplementary Fig. S4c–f), suggesting a little stronger electron-phonon coupling strength^39–41^, which could be one of the reasons for the red-shifted band from ~600 to ~632 nm, as well as other slight changes including red-shifted PLE spectra (from ~365 to ~368 nm), prolonged lifetime (from ~4.3 to ~4.8 μs), and narrower band gap (experimentally from ~3.05 to ~3.00 eV, and theoretically from ~2.43 to ~2.42 eV), shown in Fig. 2i–k and Supplementary Fig. S4g, h.

Once further increasing the temperature up to *T*_melting_ ~ 144 °C (called *T*_m_ hereafter), the sample would undergo the second thermal absorption, shown as the second step of TGA curve in Fig. 2b (mass losses are ~5% and ~3% compared to the *α*- and *β*-phase, respectively) and the second negative DSC signal in Fig. 2c. The melting liquid shows no emission property, while it becomes a transparent solid (Fig. 2f) with a bright orange-red emission but without any XRD peaks after natural quenching to room temperature (Fig. 2a, 160 °C pre-heated condition), illustrating the formation of the amorphous luminescent ETP_2_SbCl_5_ glass (G-ETP_2_SbCl_5_). Thanks to the great fluidity of the melting liquid, it could be designed into any complex 3D shapes utilizing customized silicone molds, such as a pumpkin or a model girl, as shown in Fig. 2g, indicating a potential application in the field of 3D luminescent mold.

The G-ETP_2_SbCl_5_ shows further red-shift in PL spectra, with a central wavelength of ~660 nm (Fig. 2h and Supplementary Fig. S5a, b), a broader FWHM value (*α*-0.42 eV, *β*-0.43 eV, G-0.52 eV), and a decreased PLQY of (52.6 ± 3.3)%. Additionally, the PLE spectrum of G-ETP_2_SbCl_5_ has also been extended and shows obvious red-shift with the highest peak at ~413 nm, shown in Fig. 2i. The G-ETP_2_SbCl_5_ still possesses an *E*_b_ of 231 > 25 meV, with a further increased *S* value of ~24.2 (Supplementary Fig. S5c–f), indicating a further enhanced electron-phonon coupling strength^39–41^. Furthermore, compared with the fluorescent lifetimes of *α*- and *β*-phase, the G-ETP_2_SbCl_5_ appears a double exponential decay behavior with a lifetime decreases to ~3.6 μs (Fig. 2j. This may be attributed by defects that induced by disordered organic groups and [SbCl_5_] octahedrons, which will be discussed in the following text), and its band gap further shortens to ~2.88 eV (Fig. 2k). These phenomena demonstrate the micro-structure of G-ETP_2_SbCl_5_ has been greatly transformed by thermal stimulation, and the specific [SbCl_5_] pyramids (such as disorder condition and bond length) may differ from the *α*- and *β*-ETP_2_SbCl_5_ powders.

### Investigation of structural distortion

**Fig. 3:**
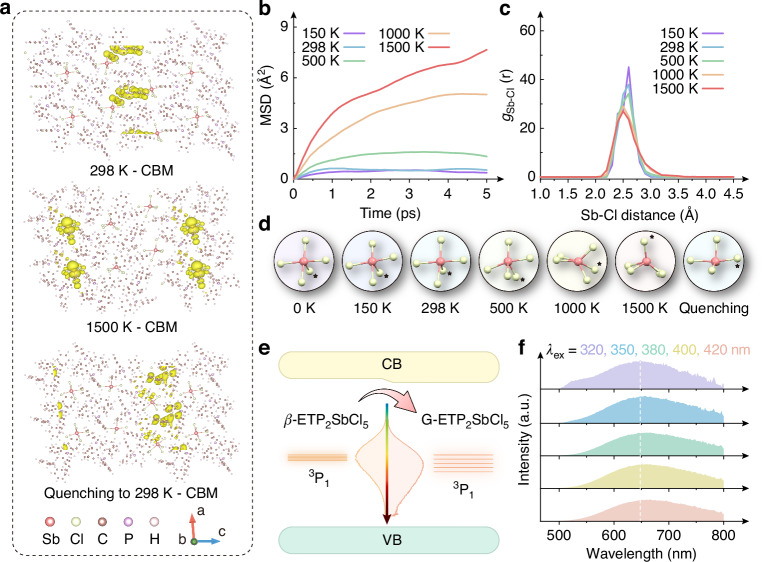
AIMD studies of thermal energy on *β*-ETP_2_SbCl_5_. **a** Crystal structures and the distributions of CBM at 298, 1500 K, and after quenching to 298 K; **b** MSD and **c** Sb-Cl RDF profiles of *β*-ETP_2_SbCl_5_ at 150, 298, 500, 1000, and 1500 K within 5 ps; **d** Simulated evolution of the [SbCl_5_] pyramid at 0, 150, 298, 500, 1000, 1500, 2000 K, and after quenching to 298 K. The focused Cl is labeled by a star for trace; **e** The concluded energy band structures for *β*- and G-ETP_2_SbCl_5_; **f** PL spectra of G-ETP_2_SbCl_5_ excited at 320, 350, 380, 400, and 420 nm

It is noted that, although G-ETP_2_SbCl_5_ could be obtained by directly heating the *α*-ETP_2_SbCl_5_ in a real operation, during which the phase transition through *β*-ETP_2_SbCl_5_ is an inevitable microscopic process. We therefore carried out the Ab initio molecular dynamics (AIMD) calculations on the *β*-ETP_2_SbCl_5_ lattice to simulate the crystal transition for deeply investigating the specific structure and luminescent mechanism of G-ETP_2_SbCl_5_. Since the simulation time (ps-scale) is far less than the actual condition (min-scale), the temperatures are set as 150–1500 K in our AIMD calculation for accelerating the molecular dynamics, thereby simulating long-term macroscopic phenomena^43,44^. The mean squared displacement (MSD) is first simulated at 150–1500 K, as shown in Fig. 3b. The curves of 150–1000 K exhibit flat trends with time, suggesting that the lattice could reach a relatively balanced state, that is, the sample may still maintain the powder state. While the MSD at 1500 K shows a monotonous increase trend in 5 ps, indicating the movement of particles may be free from the morphological constraints of powders, which could be considered as a near-molten state^45,46^.

The simulated crystal structures of *β*-ETP_2_SbCl_5_ at different temperatures are shown in Fig. 3a and Supplementary Fig. S6, S7. In the range of 0–500 K, the organic molecular units and [SbCl_5_] pyramids show a gradually distorted character with increasing temperature, along with a slightly decreased band gap (Supplementary Fig. S4g, Fig. S8, and Table S3, S4), still their overall arrangement and orientation do not change significantly (Videos S1–S3). As the temperature further increases to 1000–1500 K, the vibration of each atom is no longer limited to its original site, and the various components appear obvious distortions. For example, in one [SbCl_5_] close-up in Fig. 3d, the focused Cl atom labeled by a star symbol breaks away from the initial position and relocates to a new site at 1000 K, accompanied by similar obvious displacements of 4 other Cl atoms, resulting in a rotation and disorder of the pentagonal pyramid, and this condition could be further aggravated at 1500 K, corresponding to a further disordered lattice (Videos S4, S5). Meanwhile, the band gap dramatically decreases below 2.00 eV, and the compositions of the VBM and CBM appear qualitative changes at 1500 K, both of which are mainly composed of Sb and Cl atoms, shown in Fig. 3a and Supplementary Fig. S9a, suggesting a radical change in the electronic transition method for the melting liquid.

Besides, the FWHMs of the radial distribution function (RDF) curves for Sb-Cl bonds gradually increase with the temperature, as shown in Fig. 3c. Similar broadening properties could also be observed from the RDF curves for Sb-P and Sb-Sb bonds in Supplementary Fig. S9b, c. These phenomena demonstrate that the crystal field strength of Sb^3+^ in the molten state has wider coverage than the powder state, which lays the foundation for the broadened PL spectrum of the quenched G-ETP_2_SbCl_5_. Note that the slight peak shift towards a short distance direction may be caused by the canonical ensemble (NVT) model with constant volume, which may restrict the lattice expansion thus increasing the pressure on the lattice and indirectly affecting the peak position. In the actual condition, the lattice is supposed to expand with the temperature, which could be deduced from the slight PL blue-shift with increasing temperature (Supplementary Fig. S1i, S4d, and S5d)^42,47,48^. However, the isothermal-isobaric Ensemble (NPT) model, which maintains constant pressure, is extremely arduous on our servers. Our NVT-based AIMD aims to visualize the possible distortion of [SbCl_5_] pyramids during *β* → G transition and analyze its subsequent impacts on luminescence.

Next, the temperature is reduced to 298 K in a linear method (Supplementary Fig. S9d) for simulating the G-ETP_2_SbCl_5_. The kinetic energy of each atom gradually decreases, and the rotation of the [SbCl_5_] pyramids slows down progressively until it comes to a halt below a certain temperature threshold (see Video S6). Due to the relatively fast natural quenching process, the material could maintain a disordered structure, thereby forming the transparent G-ETP_2_SbCl_5_ instead of a recrystallized scattering solid (Supplementary Fig. S10). And the aforementioned extended coverage of the crystal field strength could be maintained, thus further increasing the ^3^P_1_ splitting degree of Sb^3+^ ions (Fig. 3e and Supplementary Fig. S11)^38^, which, combined with the narrower band gap in both theoretical and experimental profiles (Fig. 2k and Supplementary Fig. S8b, f), results in the broadened and red-shifted bands in both PL and PLE spectra (Fig. 2h, i). Under 350-420 nm excitation, the G-ETP_2_SbCl_5_ exhibits consistent PL behaviors, as shown in Fig. 3f. While, in addition to the organic emissive band at ~520 nm, a slight blue shift of the orange-emissive band could also be observed under 320 nm excitation, which may be attributed to some local defects formed during the quenching process. These defects could also be one of the main reasons for the shortened lifetime and decreased PLQY of G-ETP_2_SbCl_5_.

### Characteristics and performance of LSCs

**Fig. 4:**
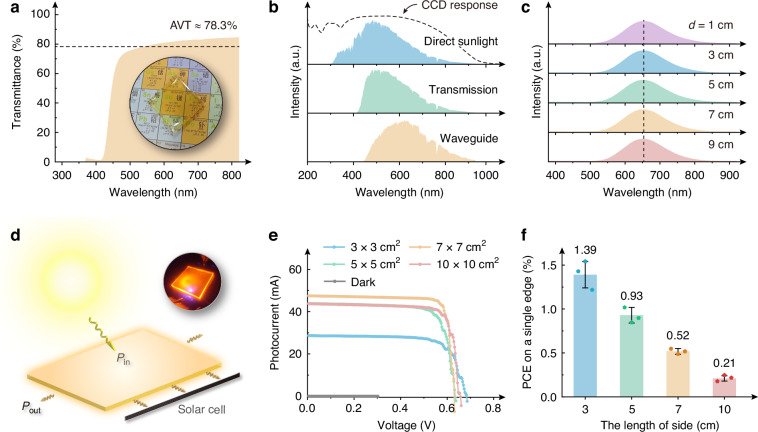
Characterizations for the G-ETP_2_SbCl_5_ and the as-fabricated LSCs. **a** Transmission spectrum of G-ETP_2_SbCl_5_. The inset is the digital photograph under indoor white light for exhibiting the transmittance effect of G-ETP_2_SbCl_5_; **b** Comparison among the spectra of direct sunlight, transmission, and waveguide light. The spectra were obtained by a charge coupled device (CCD) detector, of which the response curve is shown as the dashed line; **c** PL spectra after 1, 3, 5, 7, and 9 cm waveguide of G-ETP_2_SbCl_5_, excited by a 405 nm laser (approximately 57.2 mW cm^–2^); **d** Schematic for G-ETP_2_SbCl_5_-based LSC. The inset is the photograph of the G-ETP_2_SbCl_5_ under a vertical excitation of 395 nm; **e** *I*-*V* curves and **f** PCE values on single edges of the G-ETP_2_SbCl_5_-based LSCs with different sizes

The G-ETP_2_SbCl_5_ is then shaped into square glasses with 3, 5, 7, and 10 cm side lengths and ~0.5 cm thickness (Supplementary Fig. S12a, b) with the assistance of silicone molds. As shown in Fig. 4a, the G-ETP_2_SbCl_5_ exhibits great average visible transmittance (AVT, see Formula 3, ref. ^49^. The *T*(*λ*) is the transmittance of the glass. AM1.5 G(*λ*) represents the standard solar spectrum, and the *V*(*λ*) is the vision function curve, shown in Supplementary Fig. S12c) of ~78.3%, which could greatly guarantee the characteristics of transparent glass. On the basis that the PL range meets the external quantum efficiency (EQE) response of Si cells, the G-ETP_2_SbCl_5_ with a broader PLE band shows remarkable absorption performance for the ultraviolet (UV) light (~300–420 nm, Fig. 4a and Supplementary Fig. S12d, e), thus near-zero UV light could be detected in the solar spectrum after transmission (Fig. 4b). Then the correlated color temperature of the sunlight could be decreased from ~7556 K to ~5616 K (Supplementary Fig. S12f), leading to a softer warm-white transmission light for indoors. This fascinating property endows it with a competitive ability to block UV radiation therefore adequately protecting the human eyes and skin in practical LSC applications. On the other hand, it could also suppress the irreversible performance degradation of solar cells by UV radiation^50^.

3 $${AVT}=\frac{{\int }_{380\,{nm}}^{780\,{nm}}T\left(\lambda \right)\times {AM}1.5\,G\left(\lambda \right)\times V\left(\lambda \right)d\lambda }{{\int }_{380\,{nm}}^{780\,{nm}}{AM}1.5\,G\left(\lambda \right)\times V\left(\lambda \right)\,d\lambda }$$

Thanks to the effective waveguide behavior, the STE emission of Sb^3+^ could be conducted to the edges (Supplementary Fig. S13a–c), with the measured PLQY_edge_ values of 41.7% on 3 × 3 cm^2^ (Table 1). Although it gradually decreases with the lateral size due to the inevitable photon escape (Supplementary Fig. S13d), the PLQY_edge_ of the 7×7 cm^2^ glass could still maintain 30.4%, which is ~57.8% of total G-ETP_2_SbCl_5_, demonstrating a reliable edge output performance (The PLQY measurement for 10×10 cm^2^ glass is not available because of the size limitation of the integrating sphere). Furthermore, due to the large Stokes shift of ~240 nm, nearly no overlap could be observed between PL and PLE spectra, therefore the G-ETP_2_SbCl_5_ glass exhibits consistent spectra under a 405 nm UV laser without obvious self-absorption even with 1-9 cm of the optical waveguide distance, shown as “*d”* in Fig. 4c and Supplementary Fig. S13d. Besides the PL, a part of the sunlight could also be conducted to the edges. Hence, the resulting waveguide spectrum represents a combination of the emission from G-ETP_2_SbCl_5_ material that is excited by the UV portion of sunlight, along with the direct waveguide of the visible to near-infrared region of the solar spectrum (Fig. 4b, bottom).

**Table 1:** The parameters of the as-fabricated LSCs

Size (cm^3^)	PLQY_edge_ (%)	Single *I*_LSC_ (mA)	FF (%)	Single PCE (%)	Total PCE (%)	*η*_opt._ (%)
3 × 3 × 0.5	41.7	28.2	73.5	1.39 ± 0.16	5.56	32.5
5 × 5 × 0.5	35.9	43.8	77.0	0.93 ± 0.09	3.72	17.9
7 × 7 × 0.5	30.4	47.5	76.5	0.52 ± 0.03	2.08	9.9
10 × 10 × 0.5	--	43.7	77.4	0.21 ± 0.02	0.84	5.2

The G-ETP_2_SbCl_5_-based LSC prototypes are then fabricated by coupling commercial Si solar cells on a single edge of each G-ETP_2_SbCl_5_ glass, shown in Fig. 4d. Under the irradiation of the standard solar simulator (AM1.5 G, 100 mW cm^–2^), the 3 × 3 cm^2^ LSC reaches a short-circuit photocurrent (*I*_LSC_) of ~28.2 mA, with the highest power conversion efficiency (PCE) of (1.39 ± 0.16)%. It is noted that the *I-V* curves, output powers, and PCEs in Fig. 4e-f and Supplementary Fig. S13e are depicted by solar cells on single edges of the fluorescent glasses. It is reasonable to consider that the other three edges could output similar powers, then the total PCEs could be calculated by four folds of a single cell^51^, and the specific parameters are shown in Table 1. Among them, the optical efficiency (*η*_opt._) of the fluorescent glass, which is commonly defined as the ratio of the total number of emitted photons reaching the lightguide edge to the total number of solar photons incident onto the lightguide front surface (Formula 4, ref. ^52–54^), also achieved the highest value of ~32.5% on the 3 × 3 cm^2^ LSC (see Supplementary Table S5 and Note for performance metrics and their derivation).

4 $${\eta }_{{opt}.}=\frac{{I}_{{LSC}}\times {A}_{{edge}}}{{I}_{{PV}}\times {A}_{{LSC}}}$$

Where the *I*_LSC_ and *I*_PV_ represent the total short-circuit current of LSCs and Si solar cells, respectively, and the *A*_LSC_ and *A*_edge_ refer to the surfaces and total edge areas of LSCs, respectively. Similar to the previous reports^1,7,10^, the PCE gradually decreases with the increase of lateral size, and it is not only affected by the decreased *η*_opt._ induced by photon escape, but also by the declined PCE_PV_ of Si solar cells in larger areas (Supplementary Fig. S13f, g and Table S4). Nevertheless, the LSC with a large scale of 10 × 10 × 0.5 cm^3^ still maintains PCE and *η*_opt._ of ~0.84% and ~5.2%, respectively, without any optical amplifiers or extra waveguide media, which exhibits comparable performance to the previously reported LSCs (Supplementary Table S6), suggesting the feasibility of LSCs by using such fluorescent glass-formed lead-free perovskite derivatives. Additionally, as shown in Supplementary Fig. S14, we have further prepared another green emissive glass ETP_2_MnBr_4_ with a higher PLQY of ~70%, and the as-fabricated 10 × 10 × 0.5 cm^3^ ETP_2_MnBr_4_-based LSC exhibits total PCE and *η*_opt._ of ~0.72% and ~3.9%, respectively. Such a slightly lower LSC performance than that based on G-ETP_2_SbCl_5_ is probably due to the obvious self-absorption induced by the overlap of PL and PLE spectra. These extended results further prove the importance of a large Stokes shift for LSC, and on the other hand, demonstrate the great extensibility of fluorescent glass-based LSCs.

### Self-healing and recyclable repurposing

**Fig. 5:**
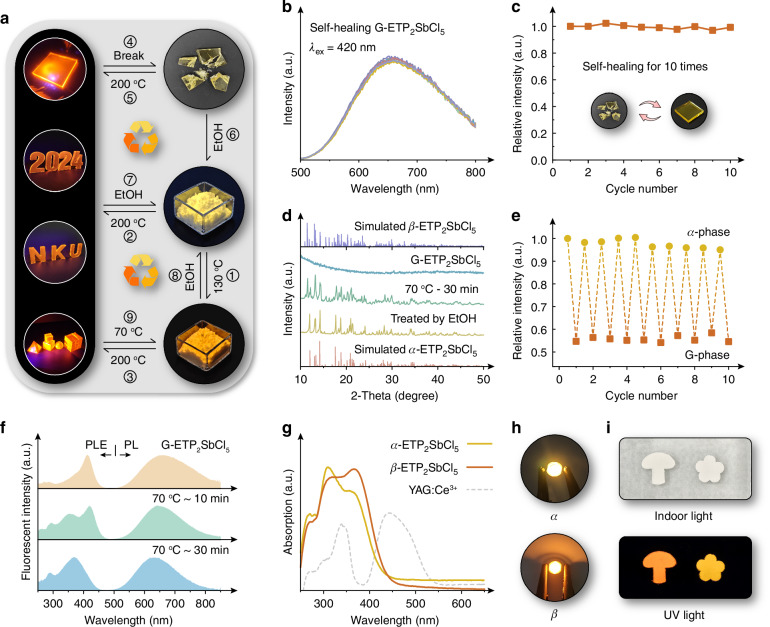
Reversible phase transitions among *α*-, *β*-, and G-ETP_2_SbCl_5_ for recyclable repurposing. **a** The schematic diagram for the reversible phase transitions among *α*-, *β*-, and G-ETP_2_SbCl_5_; **b** The PL spectra and **c** the corresponding integrated intensities of G-ETP_2_SbCl_5_ self-healing for 10 cycles; **d** XRD patterns for the products obtained by the reverse phase transition from G-ETP_2_SbCl_5_; **e** Integrated PL intensities of the products by the phase transition between *α*- and G-ETP_2_SbCl_5_ for 10 cycles; **f** PLE and PL spectra of G-ETP_2_SbCl_5_ before and after 70 °C heating; **g** Absorption spectra of returned *α*- and *β*-ETP_2_SbCl_5_. The dashed curve represents the absorption profile of commercial YAG:Ce^3+^ phosphor; Photographs of (**h**) pc-LEDs and **i** anti-counterfeiting patterns fabricated by the recycled *α*- and *β*-ETP_2_SbCl_5_ phosphors

One attraction is that this kind of fluorescent glass could realize self-healing or re-shaping even if damaged, which possesses a much lower healing temperature ( ~ 150-250 °C) than conventional inorganic glasses (e.g. > 800 °C, ref. ^55^), shown as processes 4 and 5 in Fig. 5a. The PL performance of G-ETP_2_SbCl_5_ exhibits nearly no attenuations during 10 damage-healing cycles (Fig. 5b-c). More intriguingly, the *β*- and G-ETP_2_SbCl_5_ could be restored to the initial *α*-ETP_2_SbCl_5_ powders in mass production after adding moderate ethanol, with a transition yield of >82.5%. Such a remarkable recyclable ratio may be attributed to the ethanol-assistant dissolving and recrystallization processes, shown in Fig. 5a (processes 6–8), Fig. 5d, and Supplementary Fig. S15a (inset). This reversible phase transition exhibits great fatigue resistance, and the recycled *α*-ETP_2_SbCl_5_ could still maintain ~95% of the fresh PL intensity even after 10 consecutive *α*-G transitions (Fig. 5e and Supplementary Fig. S15a). Furthermore, *β*-ETP_2_SbCl_5_ could also be re-obtained by heating G-ETP_2_SbCl_5_ at 70 °C for ~30 min (Fig. 5d), during which the recrystallization of the amorphous phase is initiated and the uniform [SbCl_5_] pyramids are re-produced (Fig. 5f), corresponding to the transition from a transparent glass to a non-transparent scattering solid.

Based on the aforementioned results, we have successfully demonstrated the reversible phase transitions among *α*-, *β*-, and G-ETP_2_SbCl_5_ (Fig. 5a), which significantly facilitates the resource recovery and repurposing even if the LSCs are damaged or decommissioned. The recycled phosphors still retain near-unity PLQYs and exhibit remarkable absorption intensities, surpassing that of the commercial YAG:Ce^3+^ (PLQY ~ 80-85%, Fig. 5g). These features position the recycled phosphors for further utilization in a variety of applications, such as phosphor converted LEDs (pc-LEDs), anti-counterfeiting, and luxury decoration, etc., on par with freshly synthesized phosphors (Fig. 5h, i and Supplementary Fig. S15b–d).

## Discussion

The stability of the G-ETP_2_SbCl_5_ is also evaluated in this work, shown in Supplementary Fig. S16, S17. Under sunlight irradiation in air, the G-ETP_2_SbCl_5_ begins to show slight recrystallization by the 10th day, and it becomes more pronounced by the 20th day (Supplementary Fig. S16a), which could be attributed to the influence of the humidity. To improve its air-stability, we have further fabricated the laminated G-ETP_2_SbCl_5_ glass by quenching G-ETP_2_SbCl_5_ between two quartz slides, as shown in Supplementary Fig. S16b. The laminated glass shows consistent transmittance spectra after exposure to sunlight in air for 20 days, without any significant recrystallization. As shown in Supplementary Fig. S16c, d, the laminated G-ETP_2_SbCl_5_ exhibits similar PL and PLE spectra after sunlight irradiation in air for 20 days, with its PLQY remaining virtually unchanged. On the other hand, this laminated approach could also prevent direct mechanical impact, thereby addressing the issue of fragile in practical applications, which is therefore considered as an effective engineering optimization solution. Furthermore, the G-ETP_2_SbCl_5_ also exhibits a reliable stability under long-term mild heating at 40-50 °C (Supplementary Fig. S17), which make it a candidate for future recyclable LSC application.

In summary, this study has presented the preparation of hybrid lead-free perovskite derivatives ETP_2_SbCl_5_, which exhibits reversible *α*-, *β*-, and G-phase, exhibiting remarkable PLQYs of (97.2 ± 2.6)%, (98.5 ± 3.2)% and (52.6 ± 3.3)%, respectively. Through AIMD and DFT simulations, we have demonstrated the disordered [SbCl_5_] pyramids with more complex Sb-Cl bonds and shortened band gap of G-ETP_2_SbCl_5_. These changes result in broadened and red-shifted PL and PLE bands, enabling it to effectively block the entire UV region (~300–420 nm) of sunlight for STE luminescence. The inaugural series of self-healing G-ETP_2_SbCl_5_-based LSCs have achieved the highest PCE and *η*_opt._ of ~5.56% and ~32.5%, respectively, on the 3 × 3 × 0.5 cm^3^ glass. Despite a decrease in PCE with increasing surface area, such novel fluorescent glass -based LSC prototypes, devoid of additional waveguide medium, have demonstrated performance on par with prior reports. Moreover, the potential for repurposing G-ETP_2_SbCl_5_ has been validated through the successful fabrication of pc-LEDs and anti-counterfeiting patterns by using recycled *α*- and *β*-ETP_2_SbCl_5_ phosphors from LSCs, underscoring its role in sustainable energy solutions for the low-carbon era.

## Materials and methods

### Raw materials

Ethyl-triphenylphosphonium chloride (ETPCl, 98%), Ethyl-triphenylphosphonium bromide (ETPBr, 98%), SbCl_3_ (99.9%), and MnBr_2_·H_2_O (98%) were purchased from Macklin and were used directly for synthesis without further purification.

### Preparation of *α*-ETP_2_SbCl_5_ powder

To synthesize 1 mmol of *α*-ETP_2_SbCl_5_ powder, 2 mmol of ETPCl (0.6536 g) and 1 mmol of SbCl_3_ (0.2281 g) were weighed and transferred into a tube. Subsequently, 5 mL of ethanol was added to the mixture. The reaction mixture was vigorously stirred for approximately 5 minutes, with intermittent ultrasonic treatment (*e.g*. ultrasonication for 30 s after every 60 s of stirring) to facilitate the reaction process, resulting in the formation of a homogeneous target product.

### Preparation of *β*-ETP_2_SbCl_5_ powder

Heating the as-synthesized *α*-ETP_2_SbCl_5_ powder at 130 °C for 10 min, results in the gradual color change from white to pale yellow, suggesting the formation of *β*-ETP_2_SbCl_5_ powder.

### Preparation of G-ETP_2_SbCl_5_ glass

Heating the as-synthesized *α*- or *β*-ETP_2_SbCl_5_ powder to 200 °C for 30 min results in the complete melting of the powders liquid, which was poured into a silicone mold with a specific shape and quenched naturally to room temperature. The transparent G-ETP_2_SbCl_5_ could be obtained after completely quenching.

### Reverse phase transition from *β*- to *α*-ETP_2_SbCl_5_

Mixing moderate ethanol with the *β*-ETP_2_SbCl_5_ sample (*e.g*. 5 mL for 1 g of sample, stirring for 1 min) drives the phase transition from *β*- to *α*-ETP_2_SbCl_5_.

### Reverse phase transition from G- to *α*-ETP_2_SbCl_5_

Mixing moderate ethanol with the G-ETP_2_SbCl_5_ sample (*e.g*. 5 mL for 1 g of sample) and vigorously stirring the mixture for 30 min affords the recycled *α*-ETP_2_SbCl_5_ phosphors. It could be accompanied by grinding, for a faster reverse transition.

### Reverse phase transition from G- to *β*-ETP_2_SbCl_5_

To observe the reverse phase transition process, we heated the G-ETP_2_SbCl_5_ at 70 °C, and collected PL spectra over a period of 30 min. To achieve a rapid and complete reverse transition from G- to *β*-phase, the glass should be ground into a powder and then heated at 70 °C for 60 min or longer.

### Preparation of ETP_2_MnBr_4_ powder and glass

The preparation of ETP_2_MnBr_4_ powder is similar to the *α*-ETP_2_SbCl_5_, while the ETP_2_MnBr_4_ glass is obtained by heating ETP_2_MnBr_4_ powder at 200 °C for 20 min and quenching to room temperature.

### Fabrication of pc-LED devices

The pc-LED was fabricated by the recycled *α*-, *β*-ETP_2_SbCl_5_ phosphors and commercial 365 nm LED chip (3 W). The mass ratio of curing glue and phosphor is 5:1, and the dispensed pc-LED was irradiated by a 5 W, 395 nm light source for 30 s to complete curing.

### Characterizations

The XRD patterns were acquired utilizing an Ultima X-ray diffractometer from Rigaku Corporation, Japan. A Cu Kα X-ray source, with a wavelength of 1.5405 Å, was employed at an operational voltage and current of 40 kV and 40 mA, respectively. The diffractometer scanned samples at a rate of 10 degrees per minute. Morphological analysis and elemental mapping were conducted on the JSM-7800F (Japan), which was integrated with an EDS system for elemental distribution studies. The TGA and DSC curves were detected by a Simultaneous Thermal Analyzer from Mettler TGA/DSC3+ (Switzerland). The index of refraction was obtained by an ellipsometry from J.A. Woollam Co., Inc. RC-2.

The PLE, PL, PLQY, TRPL spectra and luminescence lifetime decay curves were recorded using a FLS-1000 spectrofluorometer (equipped with a 300 W Xenon lamp) from Edinburgh Instruments, England. For validation, a duplicate set of measurements was performed on an FS-5 spectrofluorometer (equipped with a 300 W Xenon lamp) from the same manufacturer. The measurement systems were calibrated against a commercially available YAG:Ce^3+^ phosphor, which serves as a standard with a known PLQY of ~80%. The specific PLQY values could be calculated by Formula 5, where the numerator and denominator represent the integrated emissive photons and the absorption photons of the sample, respectively. Specifically, the *I*_PL_ is the wavelength dependence of emission intensity, while the *I*_sample_ and *I*_BG_ are the excitation light intensity with and without sample, respectively^56^. For fluorescent glasses, the PLQY_edge_ values are calculated by Formula 6, where the PLQY_surface_ and PLQY_total_ represent the PLQY values of fluorescent glasses with and without black taped edges. During the measurements, the excitation light beam was controlled to be incident on the center point of the fluorescent glass surface.

5 $$P{LQY}=\frac{\int {I}_{{PL}}d\lambda }{\int {I}_{{BG}}-{I}_{{sample}}d\lambda }$$

6 $${P{LQY}}_{e{dge}}={P{LQY}}_{t{otal}}-{P{LQY}}_{s{urface}}$$

Temperature-dependent PL spectra were generated with the Aurora 4000, from GE-UV-NIR, Changchun New Industries Optoelectronics Tech. Co., Ltd, paired with a temperature control module, HCS421VXY, provided by Instec, Shanghai Hengshang Precision Instrument Co., LTD. The absorption spectra for all samples were determined using a UV-2600 spectrophotometer from Shimadzu Corporation, Japan.

The *I*-*V* characteristics and the corresponding PCEs of the fabricated LSCs were evaluated under a dual-light source solar simulator, conforming to the Wacom Class AAA standards, which simulates AM1.5 G conditions at 25 °C with an irradiance of 100 mW cm^-2^. The corresponding *I*-*V* curves were monitored using a ADCMT 6246 source meter.

For the pc-LED devices measurements, a glovebox environment was employed to prevent environmental interference. The emission spectra of the pc-LED devices were measured with an integrating sphere (FOIS-1) from Ocean Optics, connected to a spectrophotometer (QE65 Pro). The electrical current-voltage characteristics were monitored using a Keithley 2400 source meter, with measurements taken at increments of 25 mA cm^-2^. The pc-LED devices were directly characterized without any radiator.

Digital images of the samples and luminescence in this paper were all performed using a smartphone camera (vivo X200 pro mini, default mode).

### DFT calculation

The Vienna Ab-initio Simulation Package (VASP) software was used to accomplish the DFT and AIMD calculations^57–59^. The projector augmented wave (PAW) method^60^ with the Perdew-Burke-Ernzerhof (PBE) functional^61^ was selected and the plane-wave cutoff energy was set to 500 eV. Geometric structures were fully relaxed until the energy and total forces were converged to 10^-5 ^eV and 0.01 eV Å^-1^, respectively. The Brillouin zone was sampled by the Γ-centered Monkhorst-Pack method with a k-spacing of 0.04π Å^−1^ (ref. ^62^). Twenty points were inserted between every two high symmetry points for the band structure.

### AIMD simulation

AIMD calculations were implemented at 150, 298, 500, 1000, and 1500 K based on the canonical ensemble (NVT) and Nosé-Hoover thermostat algorithm^63–65^. The AIMD simulation lasted 5 ps, with a time step of 1 fs. The quenching process was based on the same step and total time setting, and the temperature was set by Formula 7.

7 $$T={T}_{{begin}}+({T}_{{end}}-{T}_{{begin}})\times \frac{{N}_{{step}}}{{N}_{{total}}}$$

Where the *N*_STEP_ is the current step, the *N*_total_ is the total step, and the *T*_begin_ and *T*_end_ represent the starting (1500 K) and ending temperature (298 K), respectively. The electronic structures of models subjected to molecular dynamics relaxation at various temperatures were all calculated. VASPKIT code was used for all post-processing analysis^66^. The nonlinear MSD curves were caused by restricted behavior different from Brownian diffusion^67^.

### Calculation of STE spectra

The delta self-consistent field (ΔSCF) method was used to calculate energies of excited states^68,69^, and ionic relaxation was further performed with fixed electron occupancy based on the ground state configuration, while spin polarization was applied. It can be described by replacing Formula 8 with Formula 9 of one spin state, where *n*(***r***) is the density, *N* is the number of total electrons, and $\psi ({\boldsymbol{r}})$ is the KS orbital of the *i*-th electron and the orbital where CBM calculated under the ground-state was located^70^. To evaluate the influence of electron-phonon coupling, finite differences with symmetry method was applied. A one-shot sampling (ZG configuration^71^) based on the structure after relaxation with ΔSCF was employed to obtain a single distorted structure under 300 K for each phase of materials. The frequency dependent dielectric matrix was finally calculated based on the got ZG configuration, as the real part of the dielectric tensor is obtained by the usual Kramers-Kronig transformation (Formula 10), and the imaginary part is determined by Formula 11, where the *c* refers to conduction band states, and *v* refers to valence band states, *u*_*c****k***_ is the cell periodic part of the orbitals at the k-point ***k***, *P* is the principle value^72^.

8 $$n\left({\boldsymbol{r}}\right)=\mathop{\sum }\limits_{i=1}^{N}{\psi }_{i}^{* }{\left({\boldsymbol{r}}\right)\psi }_{i}\left({\boldsymbol{r}}\right)$$

9 $$n\left({\boldsymbol{r}}\right)=\mathop{\sum }\limits_{i=1}^{N-1}{\psi }_{i}^{* }{\left({\boldsymbol{r}}\right)\psi }_{i}\left({\boldsymbol{r}}\right)+{\psi }_{{CBM}}^{* }{\left({\boldsymbol{r}}\right)\psi }_{{CBM}}\left({\boldsymbol{r}}\right)$$

10 $${\varepsilon }_{\alpha \beta }^{\left(1\right)}\left({\boldsymbol{\omega }}\right)=1+\frac{2}{\pi }P{\int }_{0}^{{\infty }}\frac{{\varepsilon }_{\alpha \beta }^{(2)}{(\omega }^{{\prime} }{)\omega }^{{\prime} }}{{\omega }^{{\prime} 2}{-\omega }^{2}+i\eta }{d\omega }^{{\prime} }$$

11 $${\varepsilon }_{\alpha \beta }^{\left(2\right)}\left({\boldsymbol{\omega }}\right)=\frac{{4\pi }^{2}{e}^{2}}{\varOmega }{{lim}}_{q\to 0}\frac{1}{{q}^{2}}\sum\limits_{c,v,{\boldsymbol{k}}}{2w}_{{\boldsymbol{k}}}\delta\left(\varepsilon _{c{\boldsymbol{k}}}{-\varepsilon }_{v{\boldsymbol{k}}}-{\boldsymbol{\omega }}\right)\times{\left\langle{u}_{c{\boldsymbol{k}}{\boldsymbol{+}}{{\boldsymbol{e}}}_{\alpha }q}{{{\mid}u}}_{v{\boldsymbol{k}}}\right\rangle{\left\langle{u}_{c{\boldsymbol{k}}{\boldsymbol{+}}{{\boldsymbol{e}}}_{\beta }q}{{{\mid}u}}_{v{\boldsymbol{k}}}\right\rangle}}^{*}$$

### Waveguide simulation

The two-dimensional simulation on the waves propagation in the luminescent layer was performed by COMSOL Multiphysics, in which the wave optics module was adopted. The impedance and matching boundary condition, which were generally used for the calculation of total internal reflection in a waveguide, were set in four directions. To ensure the total reflection at the interface between the luminescent layer and the air layer, the incident angle should be larger than 42^o^. For the luminance in all the directions, more than half of the light rays satisfy the total reflection condition, hence could be restricted and propagate in the above discussed waveguide. A fine rectangular mesh with an element size of one-fifteenth wavelength (*λ*/15) were used for the whole computational domain to guarantee the accuracy of numerical results.

## Supplementary information

Supplementary Information

Supplemantary Checklist

Video S1

Video S2

Video S3

Video S4

Video S5

Video S6

## Source data

Source data

## Data Availability

The data that support these findings are available from the corresponding author upon request.
