# Supplementary material for: Recyclable luminescent solar concentrator from lead-free perovskite derivative
Source: Light Sci Appl. 2025 Aug 28;14:297. doi: 10.1038/s41377-025-01973-0 (PMC12394575; doi:10.1038/s41377-025-01973-0)
Supplement: Supplementary file 1 — Supplementary Information [file 41377_2025_1973_MOESM1_ESM.docx]

**Supplementary Information for**

**Recyclable luminescent solar concentrator from lead-free perovskite derivative**

Huanxin Yang, Haolin Lu, Xuejiao Wang, Wenda Sun, Yujing Yang, Wei Xiong, Guankui Long, Jialiang Xu, Xiaodan Zhang, Mingjian Yuan, Xiyan Li*


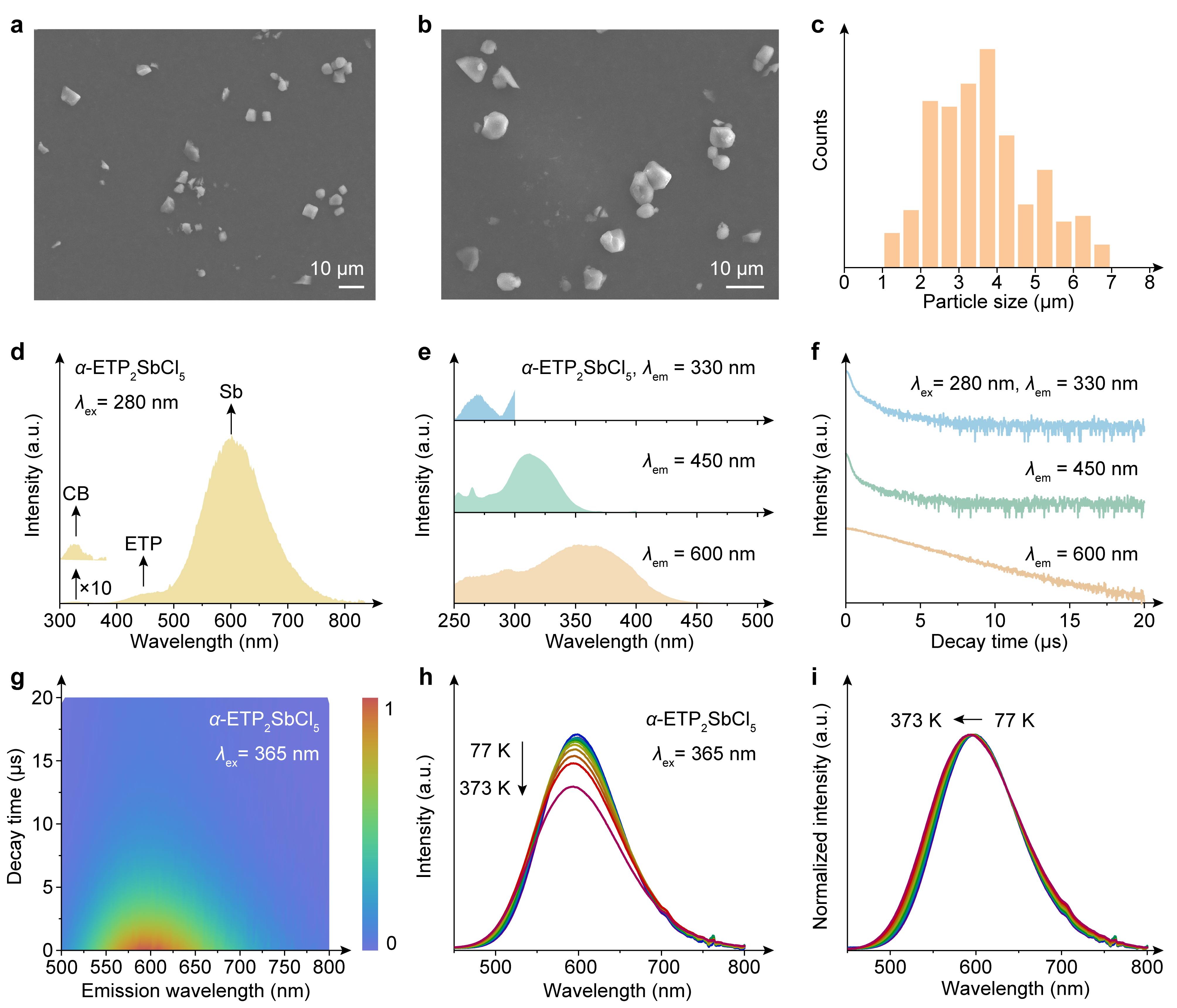


**Fig. S1. Morphology and luminescent characterizations for *α*-ETP_2_SbCl_5_ powders. (a-b)** SEM images; **(c)** Size distribution of particles; **(d)** PL spectrum excited at 280 nm; **(e)** PLE spectra monitored at 330, 450, and 600 nm; **(f)** PL decay curves monitored at 330, 450, and 600 nm; **(g)** TRPL spectra in 3D mode excited at 365 nm; **(h)** Temperature-dependent PL spectra and the **(i)** normalized spectra excited at 365 nm.


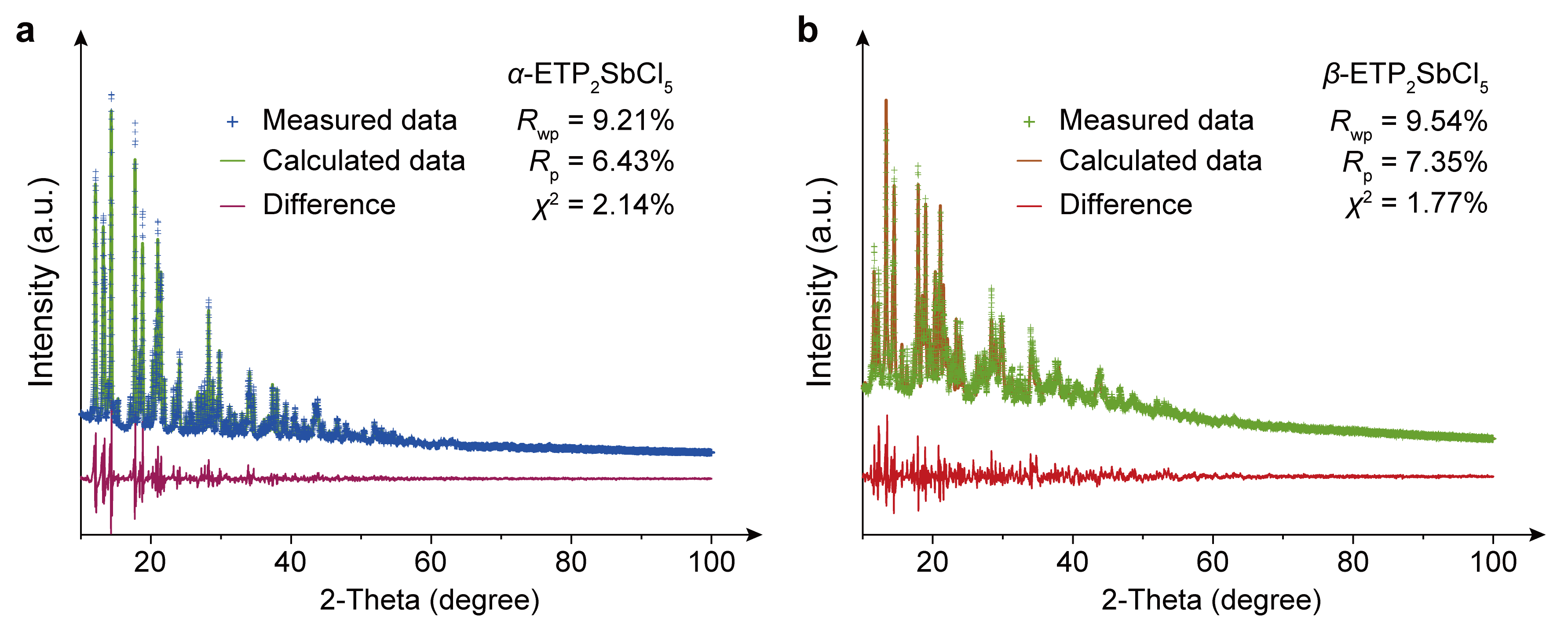


**Fig. S2. Rietveld refinement patterns. (a)** *α*-ETP_2_SbCl_5_; **(b)** *β*-ETP_2_SbCl_5_.

Through General Structure Analysis System (GSAS) software, the final refined *R*_wp_, *R*_p_, and *χ*^2^ are 9.21%, 6.43%, and 2.14%, respectively, for the *α*-ETP_2_SbCl_5_ (Fig. S2a), while they are 9.54%, 7.35%, and 1.77%, respectively, for the *β*-ETP_2_SbCl_5_ (Fig. S2b). The results show that the as-synthesized powders greatly accord with the previous reports^1-2^.


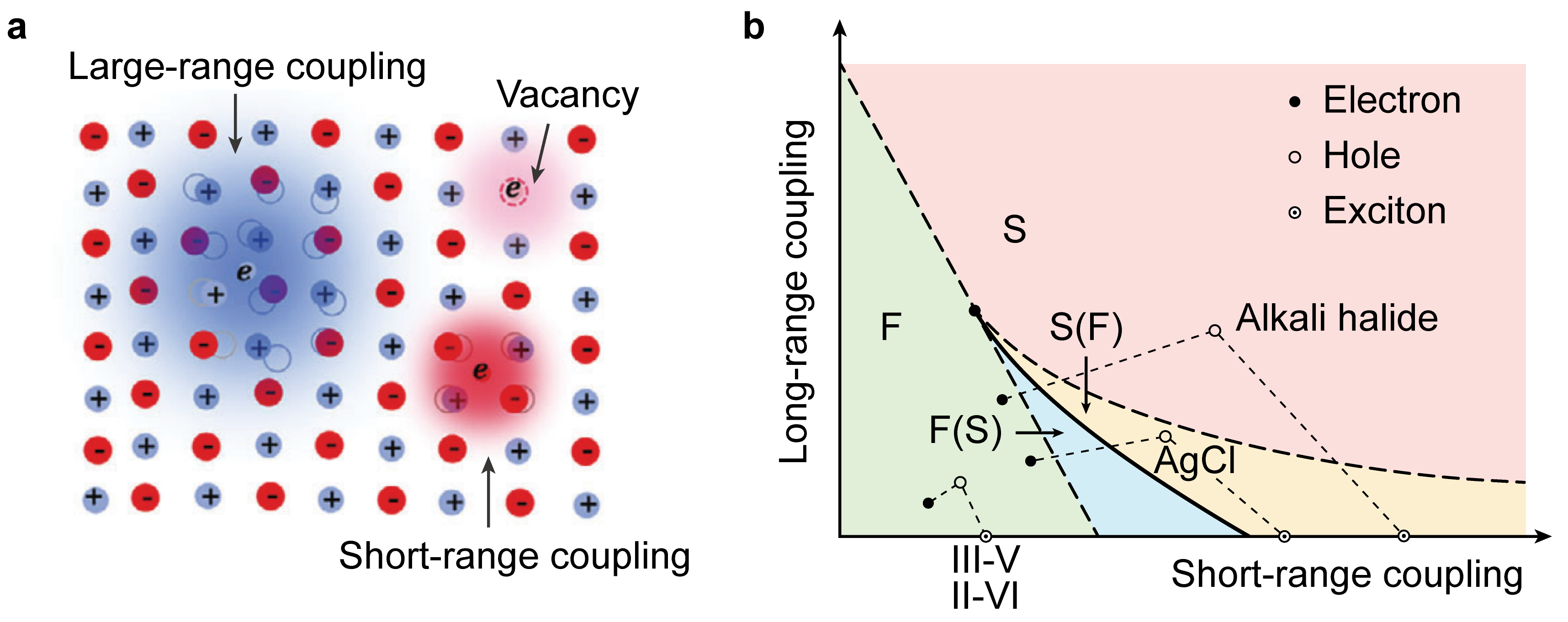


**Fig. S3. Electron-phonon coupling and self-trapped exciton. (a)** Large- and short-range coupling; **(b)** Scheme of carrier states determined by long- and short-range coupling effects. The F and S are free and self-trapped states, respectively. The F(S) represents the carriers are mainly in free state with a small portion of self-trapped state, and vice versa for the S(F).

**Note:** In this part, the Huang-Rhys factor “*S*” is processed by italic and colon for distinguishing S region in Fig. S3b.

In Fig. S3a, the large-range coupling generally involves moderate deformation extending over several unit cells. In this case, electron-phonon coupling effect is relatively weak (corresponding a small value of Huang-Rhys factor “*S*”), and the momentary trapping time is extremely short, which is expressed mainly as a slight increase of effective mass; In contrast, the short-range coupling generally involves stronger lattice deformation confined to approximately one unit cell, resulting from short-range potentials with ion displacements that are substantial fraction of the lattice constant, corresponding to a strong coupling effect and a large value of “*S*”. In this case, the momentary trapping could be for macroscopic time intervals, such as millisecond^3^. (See more specific description in ref. ^4^, pages 16-23)

The long- and short-range coupling effects generally co-exist in a specific lattice, and their strengths determine the states of carriers, shown in Fig. S3b. Generally, a material with 10 < “*S*” < 100 is generally considered as strong electron-phonon coupling for efficient STE emission, that is the S region. With the decrease of “*S*”, the carriers would undergo S(F) and F(S) states in sequence before smoothly transiting to the F state, instead of directly jumping to the F state. In recent years, the materials with 4 < “*S*” < 10 could also exhibit efficient STE emission (*e. g.* “*S*” = 7.3 in ref. ^5^, “*S*” = 5.1 in ref. ^6^, and “*S*” = 4.1 in ref. ^7^), which may be attributed to the effective S(F) or F(S) states.

In this work, the prepared *α*-ETP_2_SbCl_5_ exhibits “*S*” value of ~7.2, suggesting that its carriers may locate in S(F) region, expressing as mainly STE emission with a small portion of free exciton emission. Therefore, under 280 nm excitation, besides the ~450 nm emission from organic groups, the *α*-ETP_2_SbCl_5_ exhibit weak 330 nm (free exciton) and strong 600 nm (STE) emissions, simultaneously, which is consistent with the above analysis.


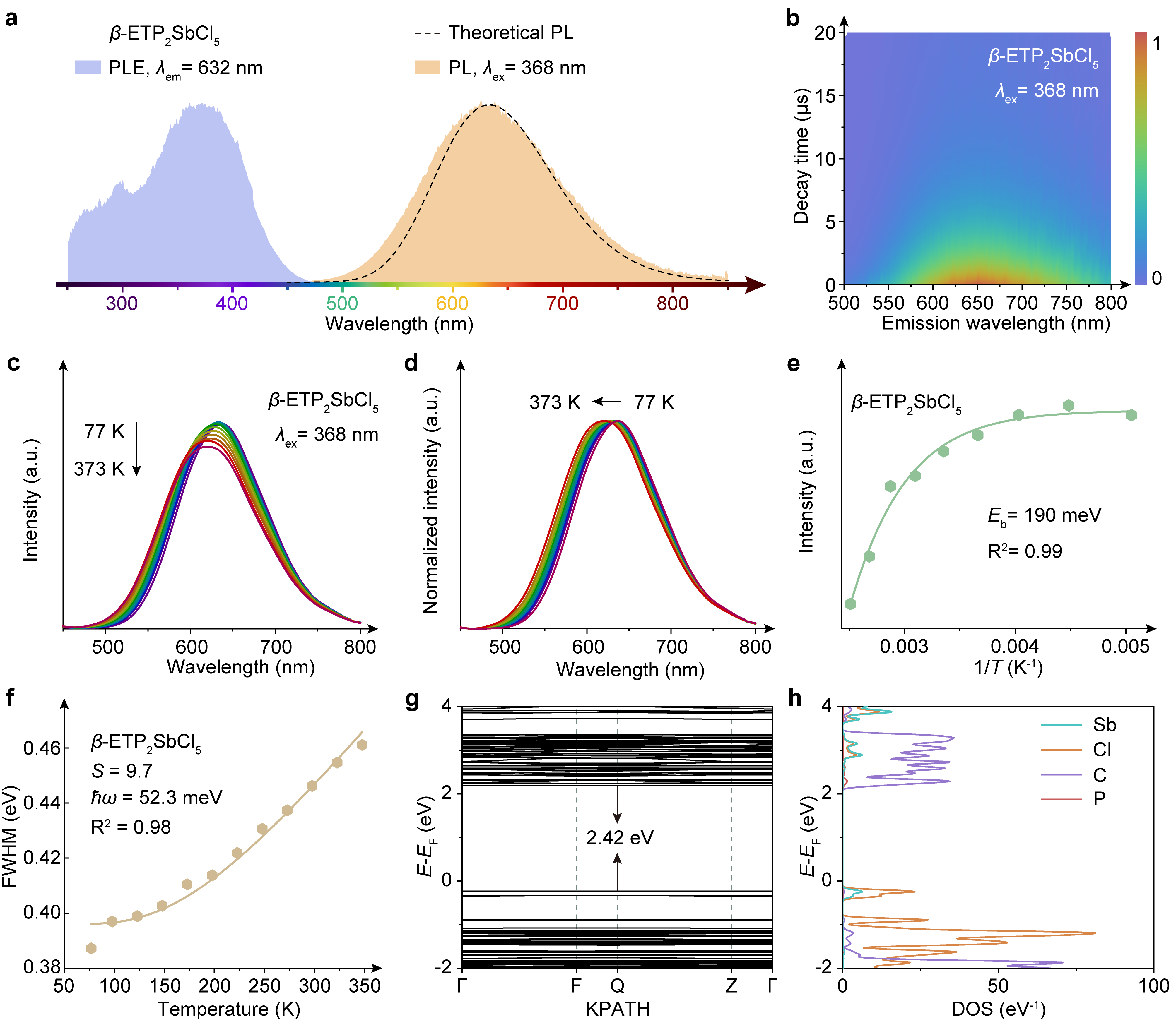


**Fig. S4. Luminescent characterizations for *β*-ETP_2_SbCl_5_ powders. (a)** TRPL spectra in 3D mode; **(b)** Temperature-dependent PL spectra and **(c)** normalized spectra excited at 368 nm; Fitting results for **(d)** *E*_b_, **(e)** *S* and *ħω* parameters; **(f)** Energy band structures and corresponding DOS profiles by DFT calculation.


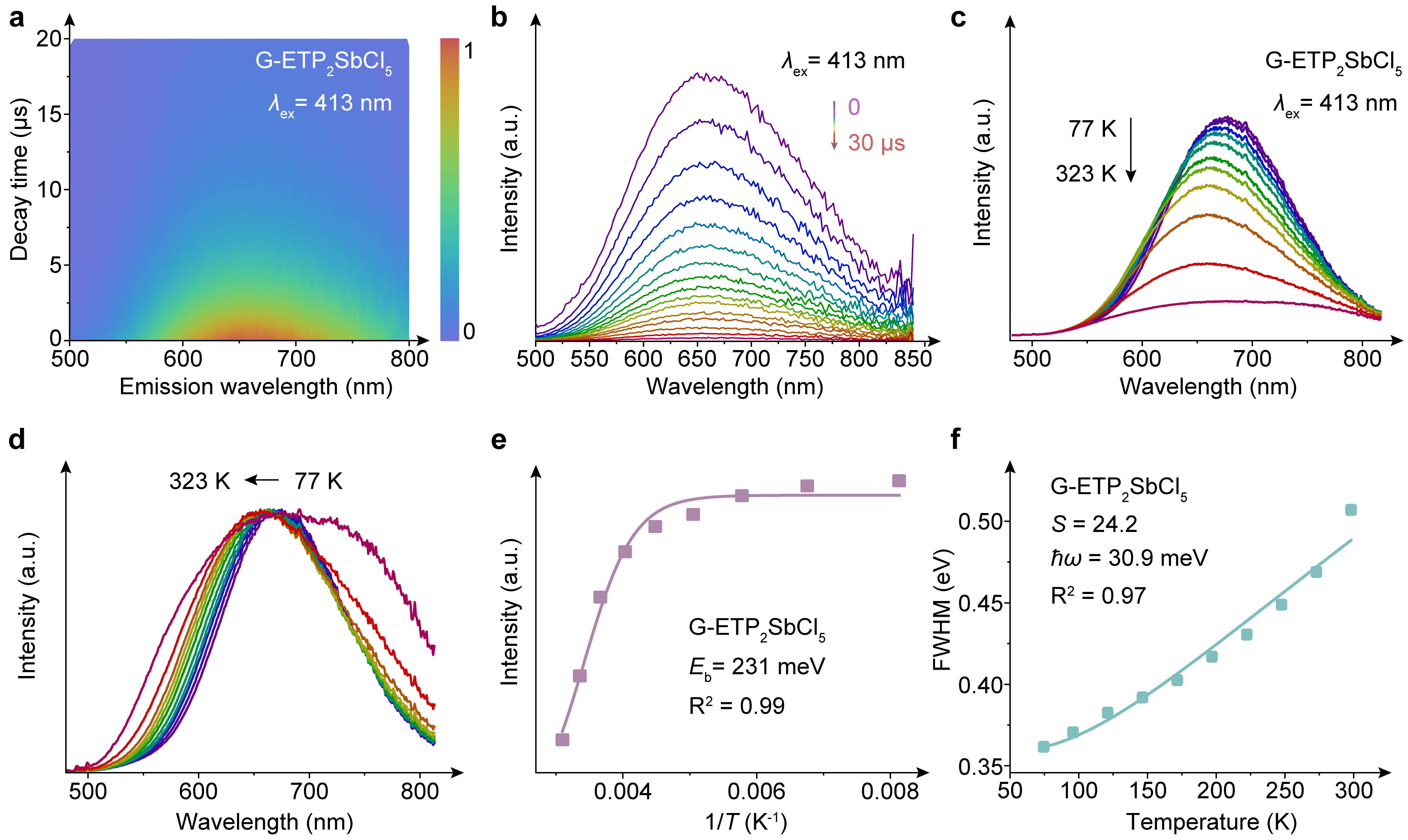


**Fig. S5. Luminescent characterizations for G-ETP_2_SbCl_5_ glasses.** TRPL spectra in **(a)** 3D and **(b)** 2D modes; **(c)** Temperature-dependent PL spectra and **(d)** normalized spectra excited at 413 nm; Fitting results for **(e)** *E*_b_, **(f)** *S* and *ħω* parameters.


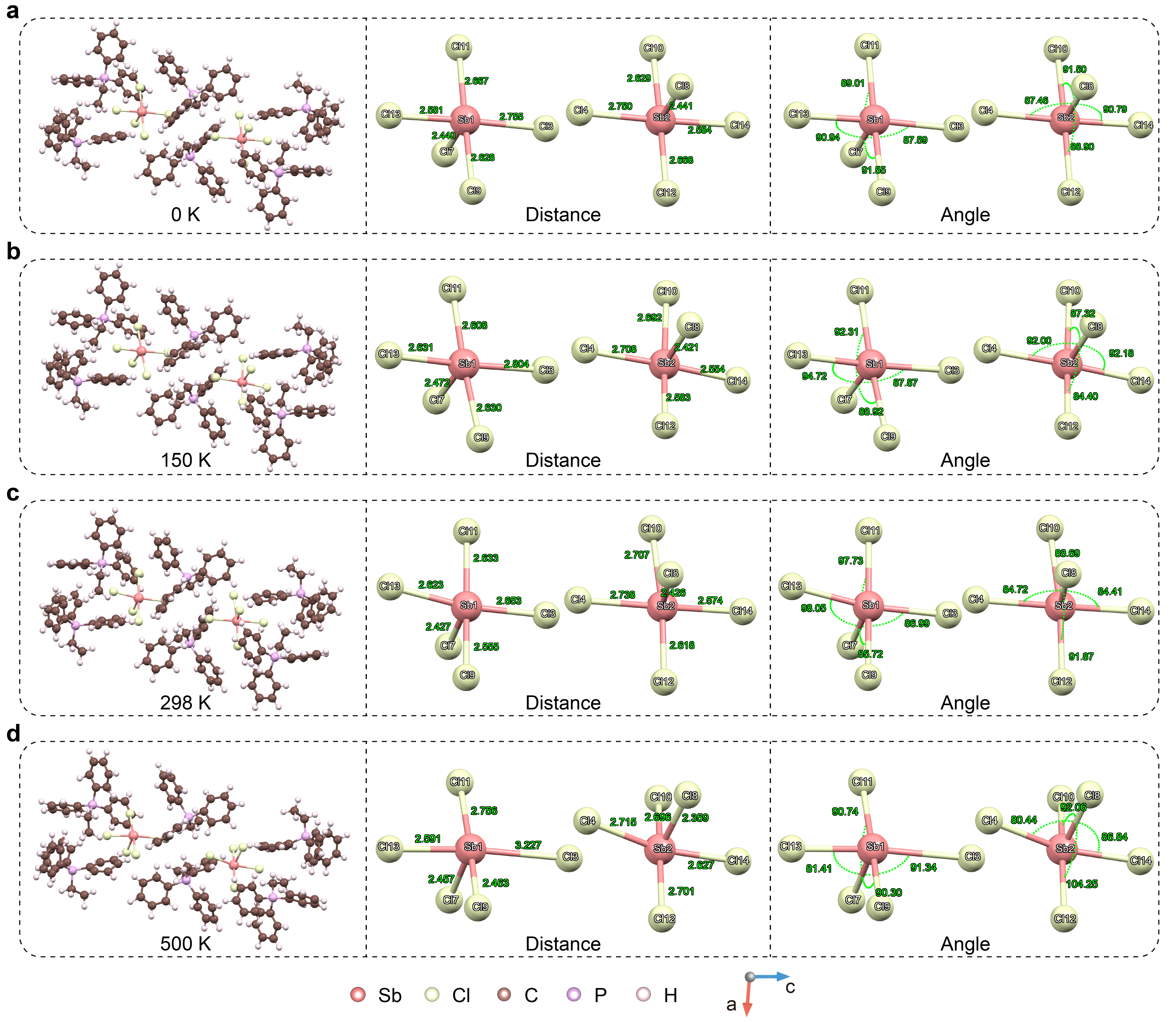


**Fig. S6. Lattices of the *β*-ETP_2_SbCl_5_ and the close-up pictures of [SbCl_5_] pyramids under various temperatures by AIMD simulation. (a)** 0, **(b)** 150, **(c)** 298, and **(d)** 500 K.


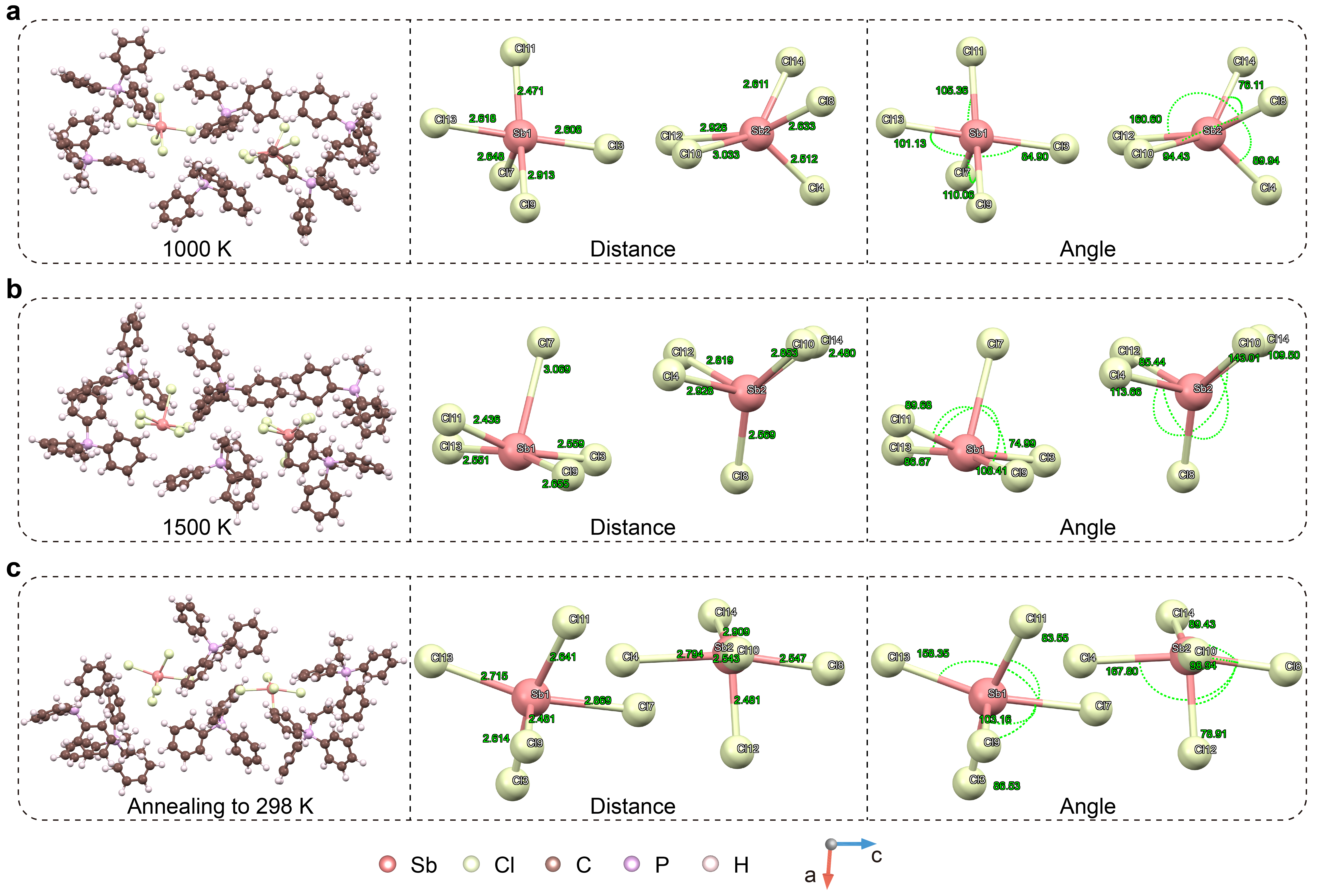


**Fig. S7. Lattices of the *β*-ETP_2_SbCl_5_ and the close-up pictures of [SbCl_5_] pyramids under various temperatures by AIMD simulation. (a)** 1000, **(b)** 1500 K, and **(c)** quenching to 298 K (transition to G-ETP_2_SbCl_5_).

**
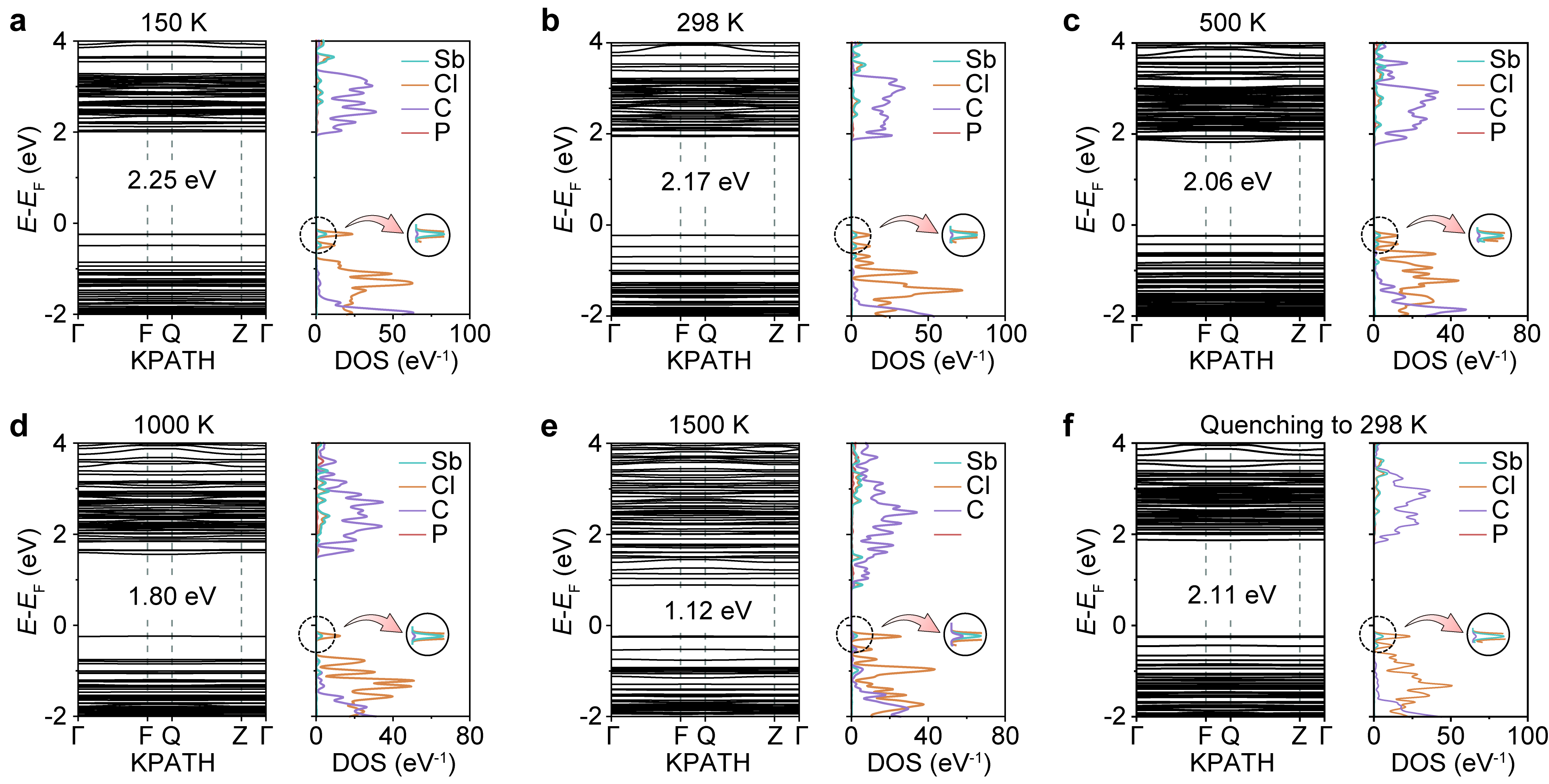
**

**Fig. S8. Energy band structures and DOS profiles of the *β*-ETP_2_SbCl_5_ under various temperatures. (a)** 150, **(b)** 298, **(c)** 500, **(d)** 1000, **(e)** 1500 K, and **(f)** quenching to 298 K (transition to G-ETP_2_SbCl_5_).

**
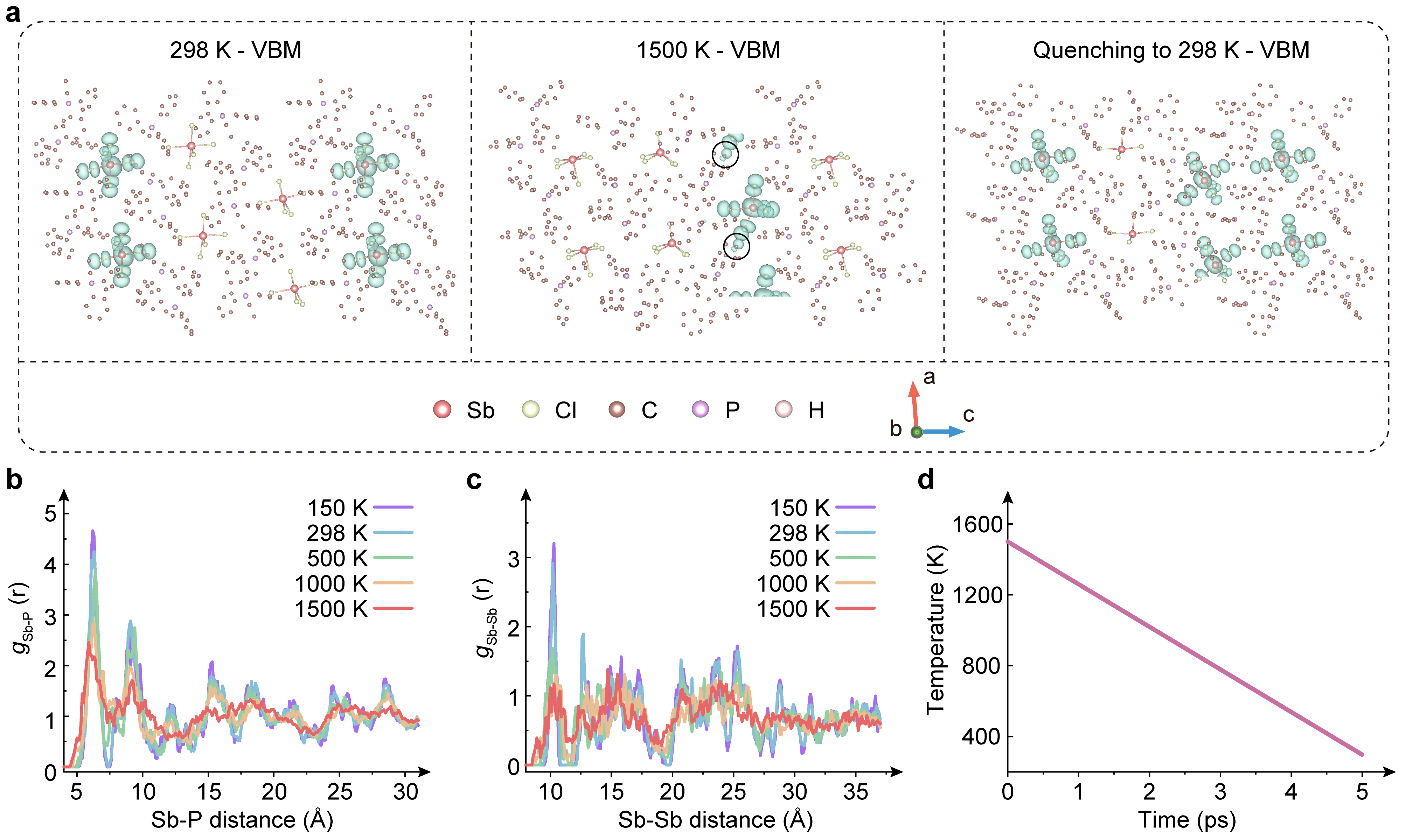
**

**Fig. S9. Lattice changes of the *β*-ETP_2_SbCl_5_ under various temperatures. (a)** Crystal structures and the distributions of VBM at 298, 1500 K, and after quenching to 298 K; **(b)** Sb-P and **(c)** Sb-Sb distance distributions; **(d)** Simulated temperature function during the quenching process.


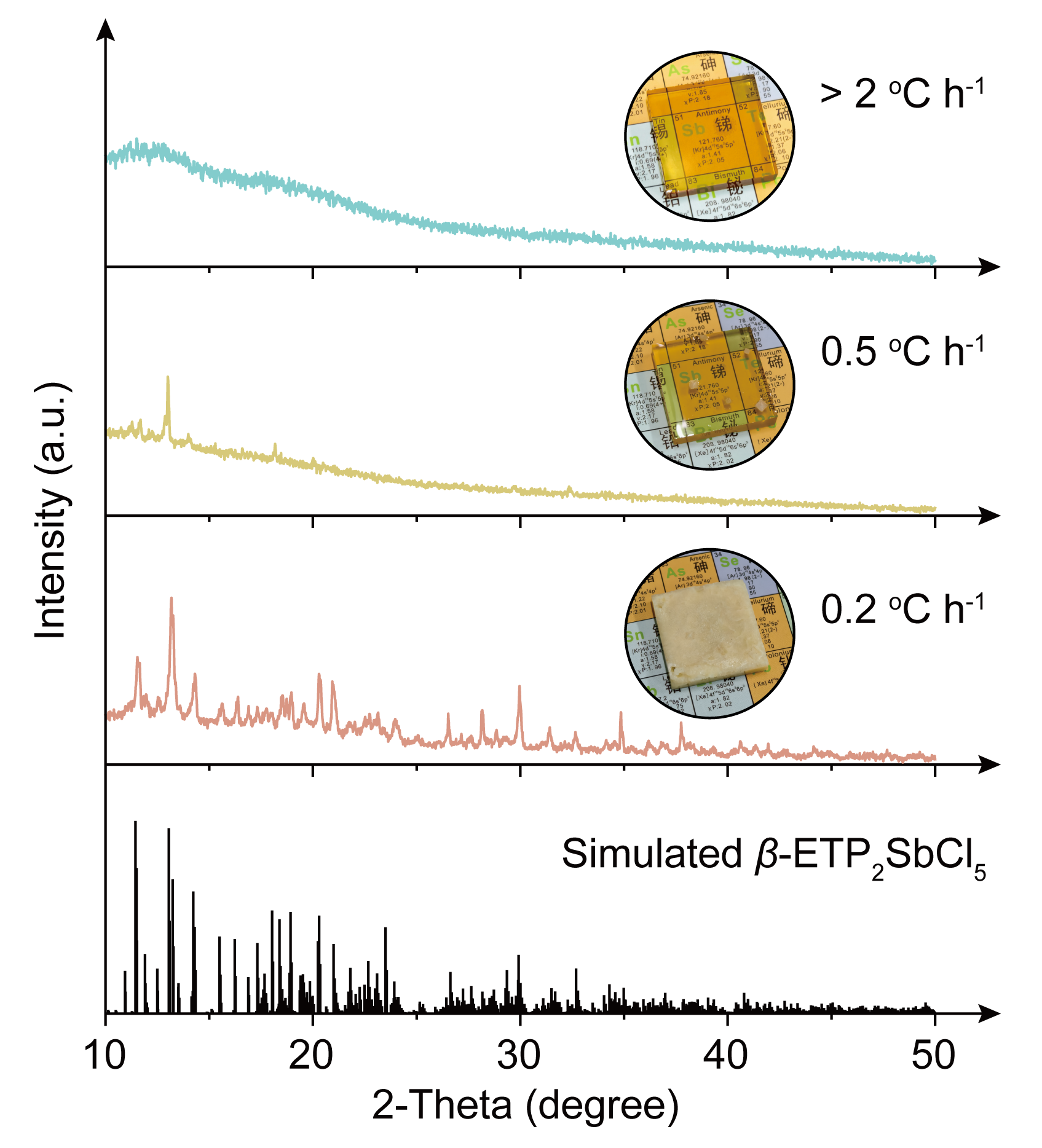


**Fig. S10. XRD patterns for the products with different cooling rates from 150 ^o^C to 30 ^o^C.** **(a)** >2 ^o^C h^-1^; **(b)** 0.5 ^o^C h^-1^; **(c)** 0.2 ^o^C h^-1^.

During the cooling process, there is a competition between solidification to amorphous phase and recrystallization to crystal phase^8^, which is primarily dependent on the cooling rate. For example, the transparent G-ETP_2_SbCl_5_ glass could be obtained with cooling rate of > 2 ^o^C h^-1^; As the cooling rate decreases to ~0.5 ^o^C h^-1^, there are some obvious non-transparent solids randomly distributed in the glass, with a slight XRD signal indicating partial crystallization; While further decreasing the cooling rate to ~0.2 ^o^C h^-1^, the product would become a non-transparent solid, with obvious XRD patterns, corresponding to the simulated diffraction of *β*-ETP_2_SbCl_5_.

**
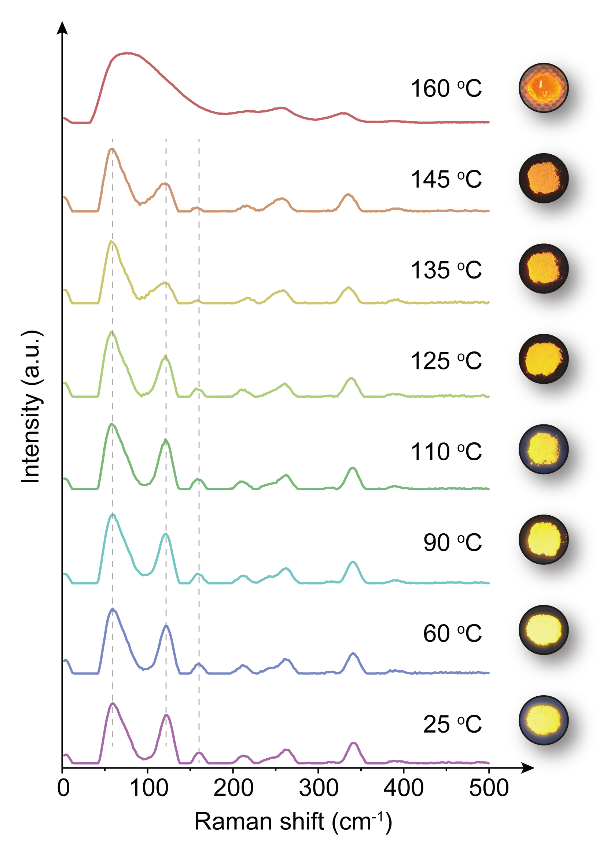
**

**Fig. S11. Raman spectra.** Raman spectra of as-synthesized *α*-ETP_2_SbCl_5_ products after being pre-treated by different temperatures for 10 min. The insets are the luminescent photographs of the samples after various heating pre-treatments, corresponding to the Fig. 2a in the main text.

The Raman peak at ~122 cm^-1^ broadens after pre-heated at 135 ^o^C, corresponding to the phase transition from *α*- to *β*-ETP_2_SbCl_5_. After pre-heated at 160 ^o^C, the G-ETP_2_SbCl_5_ is formed, and the Raman peaks at ~58, ~122, and ~158 cm^-1^ further broaden and transform into a wide band, ranging from ~32 to ~185 cm^-1^, with the highest intensity at ~76 cm^-1^, suggesting a broadened distribution of Sb-Cl bands. This phenomenon in agreement with the XRD and AIMD profiles in Fig. 2a and Fig. 3c, respectively, greatly verifying the reliability of our AIMD calculations.

**
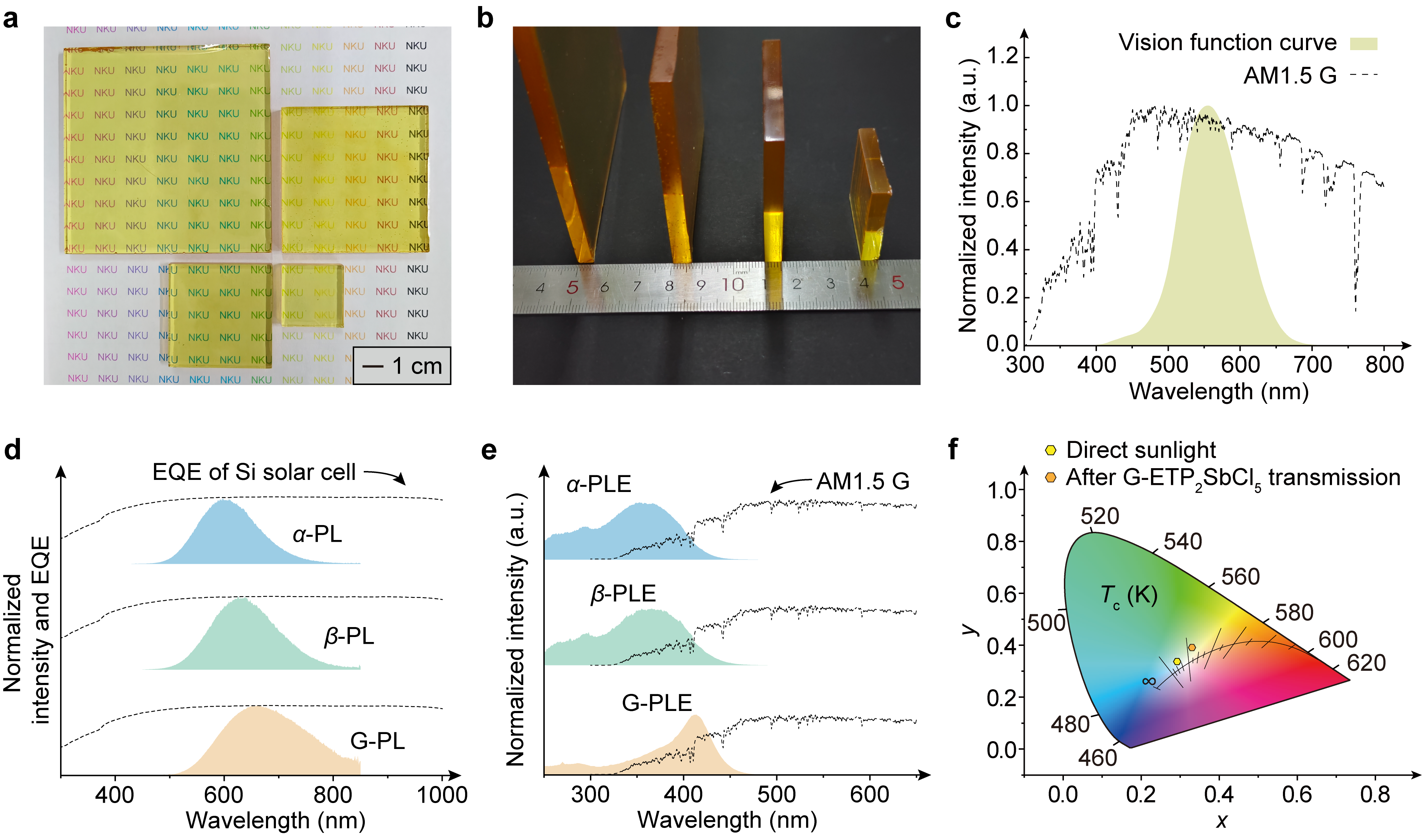
**

**Fig. S12. Characterizations for G-ETP_2_SbCl_5_ glasses and the LSC devices. (a-b)** G-ETP_2_SbCl_5_ glasses with ~3, 5, 7, and 10 cm of side lengths and ~0.5 cm of thickness. The digital photographs were pictured under indoor white light; **(c)** Vision function curve and standard solar spectrum; **(d)** PL spectra of *α*-, *β*-, and G-ETP_2_SbCl_5_. The dashed lines are the external quantum efficiency responses; **(e)** PLE spectra of *α*-, *β*-, and G-ETP_2_SbCl_5_. The dashed lines are the standard solar spectrum (AM1.5 G); **(f)** Chromaticity diagram for the sunlight before and after G-ETP_2_SbCl_5_ transmission. *T*_c_ represents the correlated color temperature.


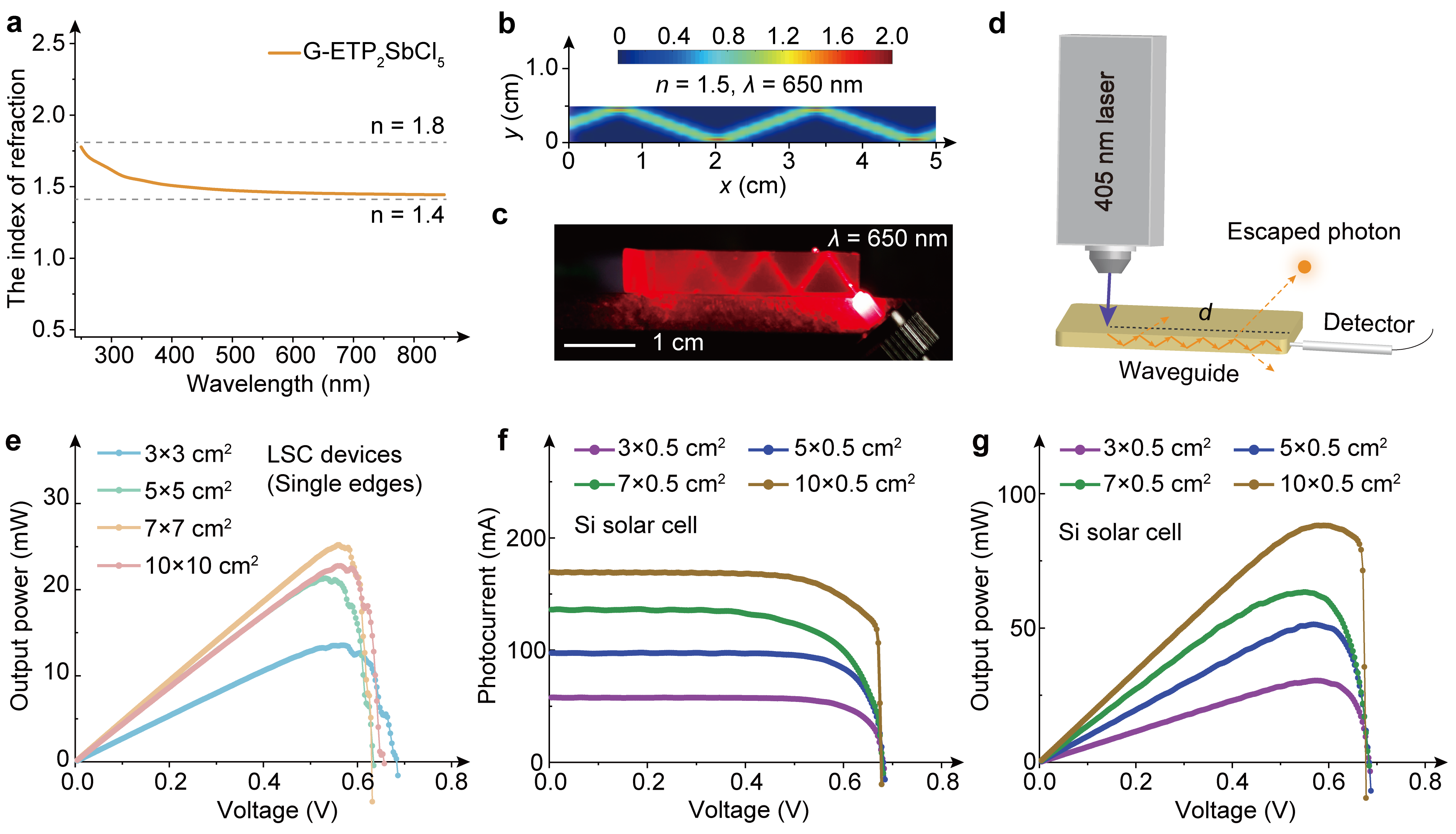


**Fig. S13. Waveguide and LSC measurements based on G-ETP_2_SbCl_5_.** **(a)** Refraction index curve; **(b)** Two-dimensional electric field distribution of the symmetric planar waveguide; **(c)** Photograph for waveguide of 650 nm laser; **(d)** Schematic diagram for waveguide distance; **(e)** Output power response of as-fabricated LSCs; **(f)** *I*-*V* curves and **(g)** output power responses of the commercial Si solar cells with lateral sizes of 3×3, 5×5, 7×7, and 10×10 cm^2^.

In Fig. S13a, the index of refraction for G-ETP_2_SbCl_5_ decreases from 1.8 to 1.4 with the increase of irradiation wavelength from 250 to 850 nm. For the light of 650 nm, the refraction index is ~1.46, which is therefore approximated as 1.5 in the following COMSOL simulation.

The three-layer structure (air-glass-air) can be regarded as a symmetrical slab waveguide. According to the waveguide theory, the fundamental mode of the planar waveguide always exists, that is, a planar waveguide at least supports the transmission of fundamental mode^9^. This viewpoint theoretically proves the feasibility of the proposed structure to guide waves.

Take the device with the side length of 5 cm and thickness of 0.5 cm as an example. Fig. S13b shows the two-dimensional electric field distribution of the symmetric planar waveguide. Multiple total reflection phenomena can be observed at the interfaces. The position with the larger intensity results from the interference enhancement of lights. The total reflection and interference enhancement are two necessary and sufficient conditions for the waveguide effect^9^, which ensure the efficient transmission of the light beam in the waveguide, and hence the guiding of luminescence to the device edges.

**
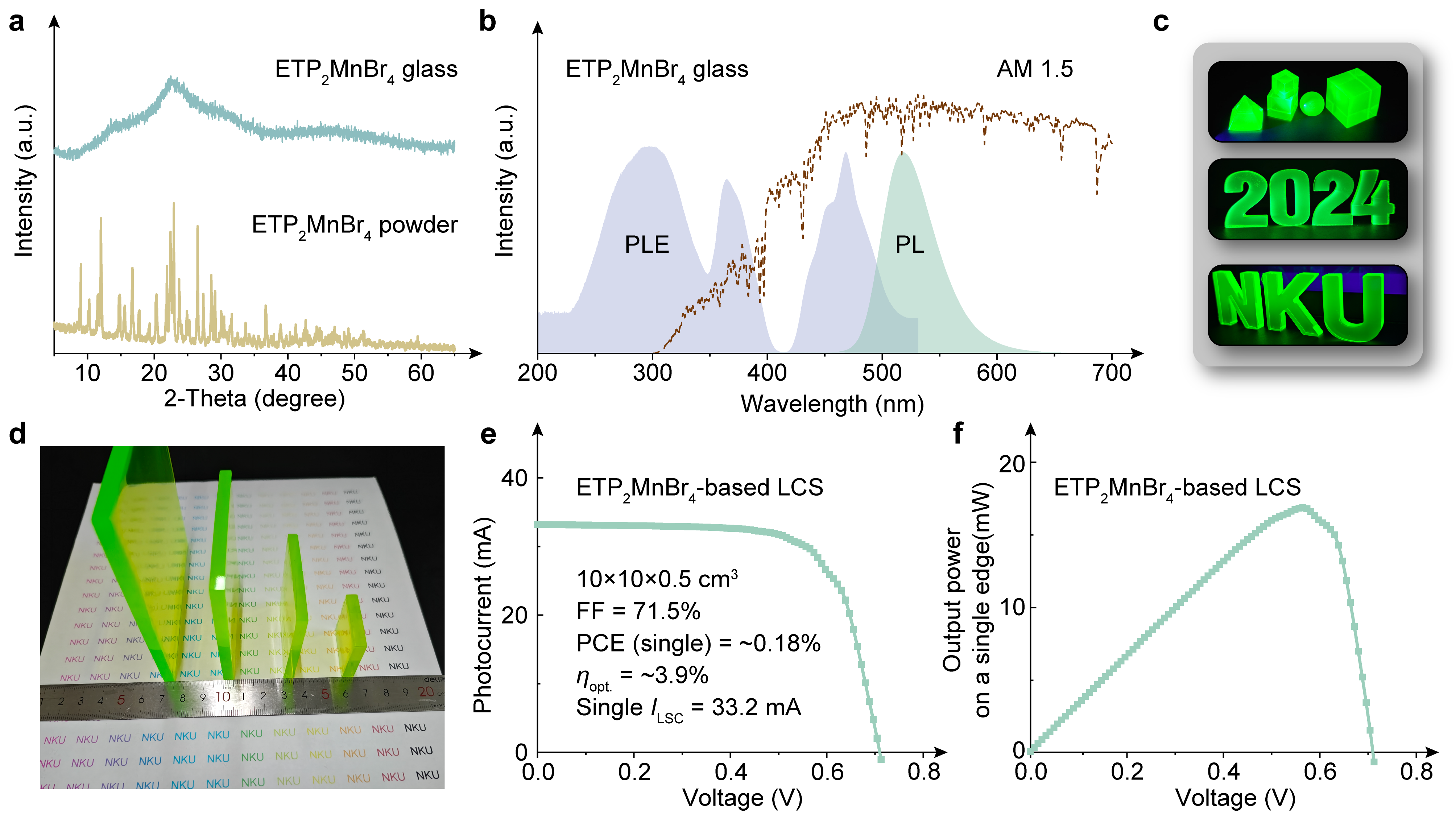
**

**Fig. S14. Characterizations of ETP_2_MnBr_4_ glass and corresponding LSC device. (a)** XRD patterns of ETP_2_MnBr_4_ powder and glass; **(b)** Normalized PL and PLE spectra of ETP_2_MnBr_4_ glass. The dashed line is the standard solar spectra (AM1.5 G); Photographs of ETP_2_MnBr_4_ glasses in **(c)** customized and **(d)** square shapes; The **(e)** *I*-*V* curve and **(f)** Output power of ETP_2_MnBr_4_-based LSC device (10×10×0.5 cm^3^).

**
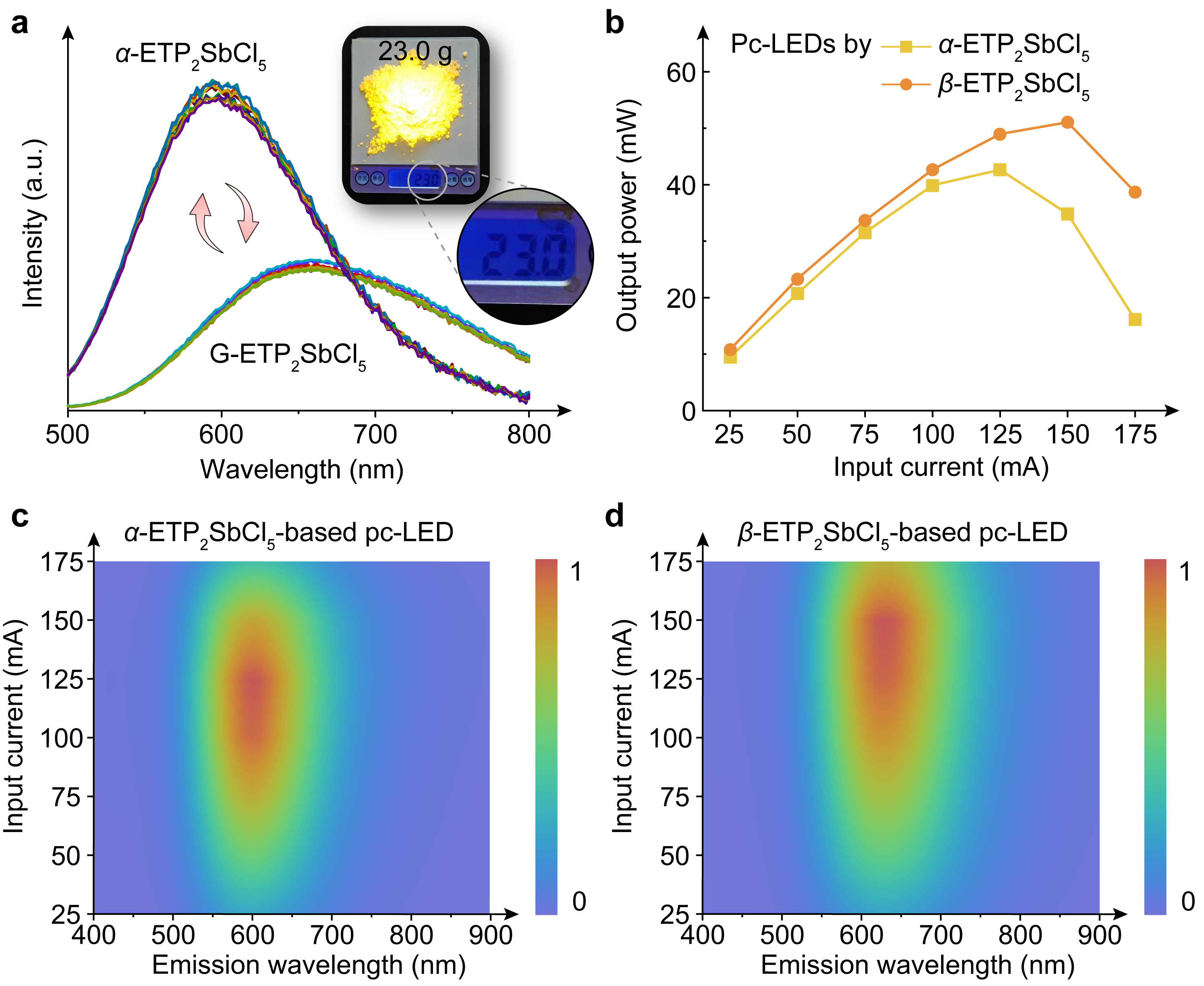
**

**Fig. S15. Reverse phase transition and corresponding performance of pc-LEDs. (a)** Consecutive phase transition between *α*- and G-ETP_2_SbCl_5_ for 10 cycles. The inset is the photograph of recycled *α*-ETP_2_SbCl_5_ powders in mass production (23.0 g); **(b)** Optical powers and **(c-d)** emission spectra in 3D modes of pc-LEDs fabricated by recycled *α*- and *β*-ETP_2_SbCl_5_ powders.


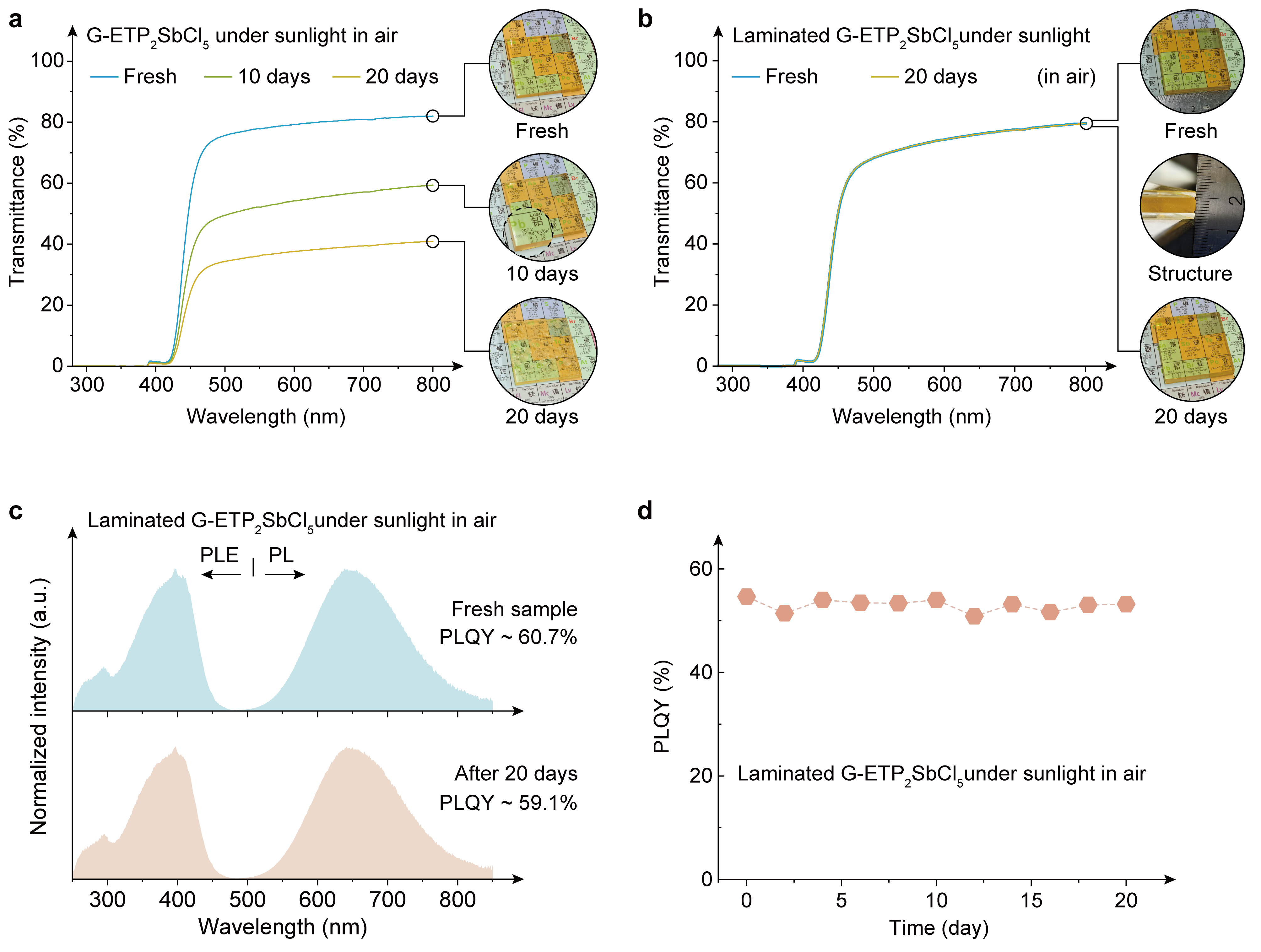


**Fig. S16. The stability of G-ETP_2_SbCl_5_ under long-term sunlight irradiation in air.** **(a)** Transmittance spectra of the G-ETP_2_SbCl_5_ at 0, 10, and 20 days. The insets are the photographs of the glass at 0, 10, and 20 days; **(b)** Transmittance spectra of the Laminated G-ETP_2_SbCl_5_ at 0 and 20 days. The insets are the photographs of the glass at 0 and 20 days, while the middle one shows the structure of the laminated G-ETP_2_SbCl_5_ glass; **(c)** PLE and PL spectra of the laminated G-ETP_2_SbCl_5_ glass before (top) and after (bottom) long-term sunlight irradiation; **(d)** PLQY values of laminated G-ETP_2_SbCl_5_ glass recorded in 20 days of sunlight irradiation.

The slight transmittance decrease of laminated G-ETP_2_SbCl_5_ glass is caused by the two quartz slides (~91% of transmittance for 300-900 nm).


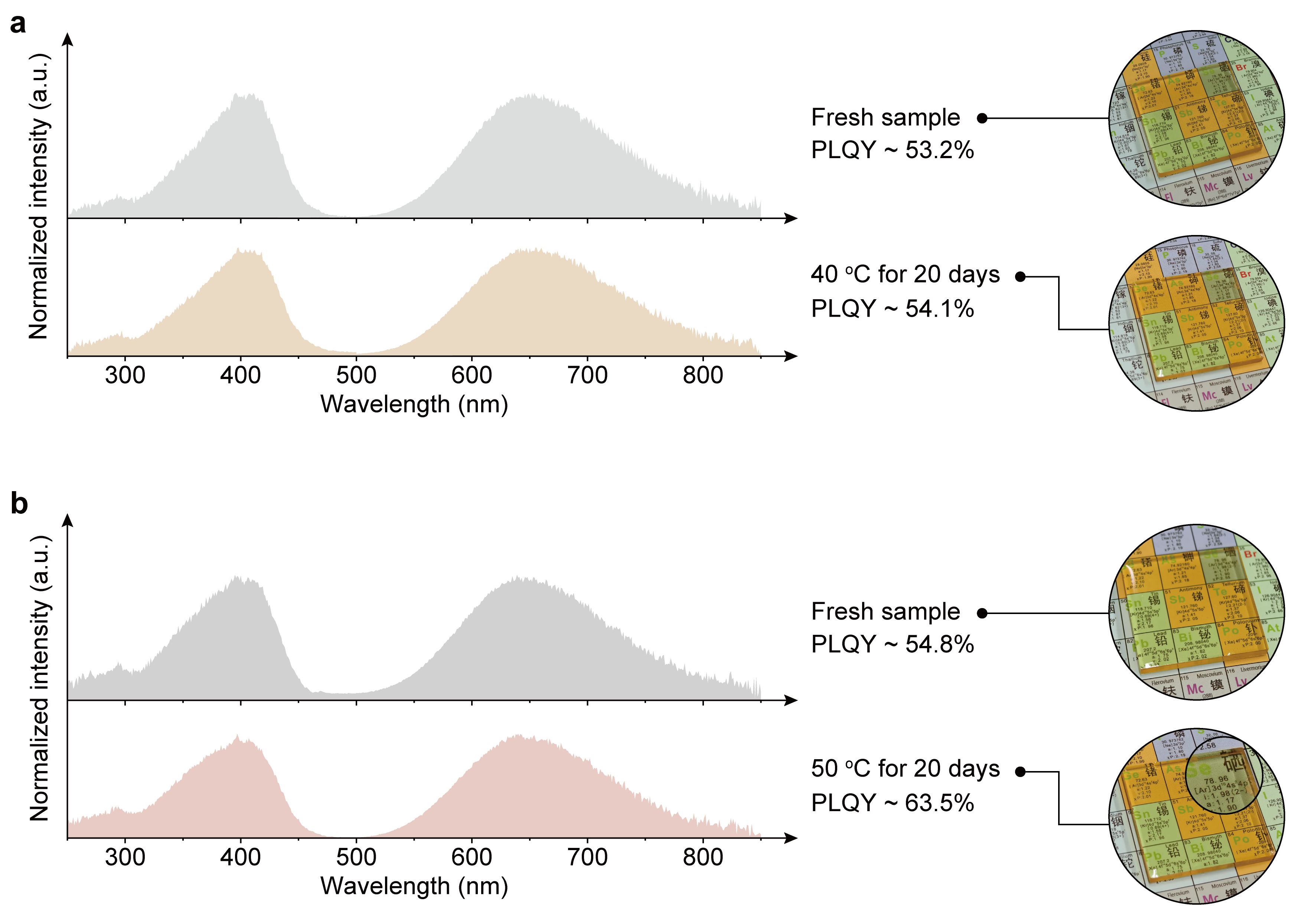


**Fig. S17. The stability of G-ETP_2_SbCl_5_ under long-term heating. (a)** PLE and PL spectra of G-ETP_2_SbCl_5_ under 40 ^o^C heating for 20 days; **(b)** PLE and PL spectra of G-ETP_2_SbCl_5_ under 50 ^o^C heating for 20 days. The insets are the photographs pictured before and after heating processes. To avoid the influence of air, the heating processes were carried out in the glove box.

The G-ETP_2_SbCl_5_ exhibits nearly no change under 40 ^o^C heating for 20 days, while it shows a slight recrystallization at the edge after 50 ^o^C heating for 20 days, which is considered as the primary reason for the slight increase of PLQY value.

**Table S1. Summary of the key LSCs and their parameters.** n.a., not applicable. (*R*=bulky cation, MA=CH_3_NH_3_, X=Cl, Br, I)

| **Candidates** | **Type** | **Fabrication Time (min)** | **Solvent** | **Chemical yield** | **Recycle** | **Ref.** |
| --- | --- | --- | --- | --- | --- | --- |
| C-dots | QDs+polymers | >900 | Need | n.a. | n.a. | ^10^ |
| Si-dots |  | >960 |  |  |  | ^11^ |
| CuInSe_2_/ZnS, Cd*_x_*Zn_1-_*_x_*S/ZnS:Mn^2+^ |  | >560 |  |  |  | ^12^ |
| *R*_2_(MA)*_n_*_-1_Pb*_n_*X_3_*_n_*_+1_ | NCs+polymers | >300 |  |  |  | ^13^ |
| Cs_4_PbBr_6_ |  | >200 |  |  |  | ^14^ |
| CsPbCl_3_:Yb^3+^ |  | >720 |  |  |  | ^15^ |
| **ETP_2_SbCl_5_** | **Self-glass** | **10-30** | **Without** | **95%** | **Yes** | **This work** |

**Table S2. Structural values for the *α*- and *β*-ETP_2_SbCl_5_.**

| **Items** | ***α*-ETP_2_SbCl_5_ (ref. ^1^)** | ***β*-ETP_2_SbCl_5_ (ref. ^2^)** |
| --- | --- | --- |
| Deposition Number | 2267284 | 2154382 |
| Space group | I 2/a (15) | P -1 (2) |
| *a* | 13.3507(4) Å | 10.21339(6) Å |
| *b* | 16.3529(5) Å | 10.28001(6) Å |
| *c* | 19.4266(6) Å | 28.72653(13) Å |
| *α* | 90^o^ | 91.1245(4)^o^ |
| *β* | 95.982(2)^o^ | 96.9622(4)^o^ |
| *γ* | 90^o^ | 100.1675(5)^o^ |

**Table S3. The lengths of Sb-Cl bonds (Unit: Å) under various AIMD temperatures.** The labels for atoms correspond to Fig. S6-S7.

|  | **0K** | **150 K** | **298 K** | **500 K** | **1000 K** | **1500 K** | **Quenching** |
| --- | --- | --- | --- | --- | --- | --- | --- |
| **Sb1-Cl3** | 2.755 | 2.804 | 2.653 | 3.227 | 2.608 | 2.559 | 2.614 |
| **Sb1-Cl7** | 2.440 | 2.472 | 2.427 | 2.457 | 2.648 | 3.069 | 2.869 |
| **Sb1-Cl9** | 2.628 | 2.630 | 2.555 | 2.463 | 2.913 | 2.655 | 2.481 |
| **Sb1-Cl11** | 2.667 | 2.608 | 2.633 | 2.756 | 2.471 | 2.436 | 2.641 |
| **Sb1-Cl13** | 2.581 | 2.631 | 2.623 | 2.591 | 2.618 | 2.551 | 2.715 |
|  |  |  |  |  |  |  |  |
| **Sb2-Cl4** | 2.750 | 2.708 | 2.738 | 2.715 | 2.512 | 2.928 | 2.794 |
| **Sb2-Cl8** | 2.441 | 2.421 | 2.426 | 2.359 | 2.633 | 2.569 | 2.547 |
| **Sb2-Cl10** | 2.629 | 2.692 | 2.707 | 2.696 | 3.033 | 2.853 | 2.543 |
| **Sb2-Cl12** | 2.668 | 2.583 | 2.618 | 2.701 | 2.926 | 2.819 | 2.481 |
| **Sb2-Cl14** | 2.584 | 2.554 | 2.574 | 2.627 | 2.611 | 2.480 | 2.909 |

**Table S4. The angles (Unit: degree) between Sb-Cl bonds under various AIMD temperatures.** The labels for atoms correspond to Fig. S6-S7.

|  | **0K** | **150 K** | **298 K** | **500 K** | **1000 K** | **1500 K** | **Quenching** |
| --- | --- | --- | --- | --- | --- | --- | --- |
| **Cl7-Sb1-Cl3** | 87.59 | 87.87 | 86.99 | 91.34 | 84.90 | 74.99 | 86.53 |
| **Cl7-Sb1-Cl9** | 91.55 | 88.92 | 85.72 | 90.30 | 110.06 | 108.41 | 103.16 |
| **Cl7-Sb1-Cl11** | 89.01 | 92.31 | 97.73 | 90.74 | 105.36 | 89.68 | 83.55 |
| **Cl7-Sb1-Cl13** | 90.94 | 94.72 | 98.05 | 81.41 | 101.13 | 86.67 | 158.35 |
|  |  |  |  |  |  |  |  |
| **Cl8-Sb2-Cl4** | 87.46 | 92.00 | 84.72 | 80.44 | 89.94 | 113.66 | 167.80 |
| **Cl8-Sb2-Cl10** | 91.50 | 87.32 | 88.69 | 92.06 | 94.43 | 143.01 | 98.94 |
| **Cl8-Sb2-Cl12** | 88.90 | 84.40 | 97.87 | 104.25 | 160.60 | 85.44 | 78.91 |
| **Cl8-Sb2-Cl14** | 90.79 | 92.18 | 84.41 | 86.84 | 76.11 | 109.50 | 89.43 |

**Table S5. The performance of Si solar cells with different lateral sizes.**

| **Lateral size (cm^2^)** | ***I*_PV_ (mA)** | **FF (%)** | **PCE_PV_ (%)** |
| --- | --- | --- | --- |
| **3×0.5** | 57.8 | 77.1 | 20.29 |
| **5×0.5** | 97.9 | 76.7 | 20.54 |
| **7×0.5** | 136.6 | 68.5 | 17.74 |
| **10×0.5** | 169.5 | 76.9 | 14.42 |

Here, we take the 3×3×0.5 cm^3^ LSC device as an example for specifically showing the calculation of *η*_opt._

(Total current of LSC) $I_{LSC}= 28.2\times4=112.8 mA$;

(Total edge area) $A_{edge}= 3\times0.5\times4 = 6 {cm}^{2}$;

(Total current of direct Si cell) $I_{PV}= 57.8\times4 = 231.2 mA$;

(Surface area of LSC) $A_{LSC}= 3\times3 = 9 {cm}^{2}$;

Therefore, $\eta_{opt.}(3\times3\times0.5 {cm}^{3})= \frac{I_{LSC}\times A_{edge}}{I_{PV}\times A_{LSC}} = \frac{112.8\times6}{231.2\times9} =32.5\%$

**Table S6. Summary of the previously reported LSCs.**

| **Candidates** | **PLQY (%)** | **Lateral size (cm^2^)** | **PCE (%)** | ***η*_opt._ (%)** | **Ref.** |
| --- | --- | --- | --- | --- | --- |
| *R*_2_(MA)*_n_*_-1_Pb*_n_*X_3_*_n_*_+1_ | 81 | 10×10 | 0.87 | 1.44 | ^13^ |
| HA_2_MA_2_Pb_3_I_10_ | 56 | 10×10 | -- | 2.0 | ^16^ |
| Cs_4_PbBr_6_ | 79 | 5×5 | -- | 1.1 | ^17^ |
| Cs_4_PbBr_6_ | 58 | 10×10 | 1.8 | 2.4 | ^14^ |
| CsPbCl_3_:Yb^3+^ | 164 | 5×5 | -- | 3.7 | ^15^ |
| CsPbCl_3_:Mn^2+^ | 10 | 20×20 | -- | 0.9 | ^18^ |
| CsPb(Br*_x_*I_1-_*_x_*)_3_ | 60 | 9×1.3 | -- | 2 | ^19^ |
| CH_3_NH_3_PbBr_3_ | 75 | 5×3 |  | 1.57 | ^20^ |
| C-dots | 65 | 15×15 | 1.13 | 2.2 | ^10^ |
| Si-dots | 46 | 12×12 | -- | 2.85 | ^11^ |
| CdSe/CdS | 45 | 21.5×1.3 | -- | 10.2 | ^21^ |
| CuInSe*_x_*S_2-_*_x_* | 40 | 12×12 | -- | 3.27 | ^22^ |
| CuInSe_2_/ZnS, Cd*_x_*Zn_1-_*_x_*/ZnS:Mn^2+^ | 78 | 15.24×15.24 (Tandem) | 3.1 | 6.4 | ^12^ |
| CuInS_2_ | >90 | 10×10 | 2.9 | 8.1 | ^23^ |
| Coumarin440 | -- | 5×5 | 0.90 | 5.71 | ^24^ |

**Note: Performance metrics (PCE, *η*_opt._) and their derivation.**

The “PCE” represents power conversion efficiency, which could be directly collected from the measurement system, and could also be calculated by:

|  | $PCE= \frac{P_{max}}{P_{in}}$ | (S1) |
| --- | --- | --- |

Where the *P*_max_ is the maximum (total) output electrical power of the edge-coupled Si cell, and the *P*_in_ is the optical power of AM1.5 G. Assuming that the output optical powers at the four edges are similar, the Formula S1 could be converted to^24^:

|  | $PCE= \frac{(4\times Single I_{LSC}){\times V}_{OC}\times FF}{J_{AM1.5 G}\times A_{LSC}}$ | (S2) |
| --- | --- | --- |

Among them, the *I*_LSC_, *V*_OC_, and FF are short-circuit current, open-circuit voltage, and fill factor, respectively, based on single edge coupled LSC device. *J*_AM1.5 G_ and *A*_LSC_ represent the optical power density of AM1.5 G and the top surface area of the LSC device, respectively.

The “***η*_opt._**” is the ratio of the optical power coming out of the edge of the LSC device (*P*_out_) relative to the optical power shooting in (*P*_in_) through the top surface, that is^14^:

|  | $\eta_{opt.}= \frac{P_{out}}{P_{in}}$ | (S3) |
| --- | --- | --- |

However, it is still a challenge to directly obtain the output optical power, which, in turn, could be indirectly calculated by PV cell. For example, *P*_out_ = *P*_LSC_/PCE_PV_, in which the *P*_LSC_ and PCE_PV_ represent the maximum (total) electrical power of LSC device and PCE of PV cell; *P*_in_ = (*P*_PV_/PCE_PV_) × (*A*_surface_/*A*_edge_), where *P*_PV_ is the maximum electrical power of PV cell (that is 4 long-strip cells) under direct shooting of AM1.5 G, while *A*_surface_ and *A*_edge_ are the areas of top surface and total edges. Then, the Formula S3 could be transformed into^24^:

|  | $\eta_{opt.}= \frac{P_{LSC} / {PCE}_{PV}}{(P_{PV} / {PCE}_{PV}) \times(A_{surface} / A_{edge})}= \frac{P_{LSC} \times A_{edge}}{P_{PV} \times A_{surface}}$ | (S4) |
| --- | --- | --- |

After substituting *P* = *I*_SC_ × *V*_OC_ × FF into the Formula S4 (*V*_OC_ and FF is consistence for the same PV cell), we can further obtain^18,25^:

|  | $\eta_{opt.}= \frac{(4 \times{Single I}_{LSC}) \times V_{OC} \times FF \times A_{edge}}{(4 \times Single I_{PV}) \times V_{OC} \times FF \times A_{surface}}= \frac{(4 \times Single I_{LSC}) \times A_{edge}}{(4 \times Single I_{PV}) \times A_{surface}}$ | (S5) |
| --- | --- | --- |

Where the (4×Single *I*_LSC_) and (4×Single *I*_PV_) are total currents of LSC devices and PV cells, respectively.

It is noted that, the Formulas S4-S5 only consider the influence of optical power, while ignores the EQE response to wavelength, thus possibly inducing a little increase of *η*_opt._(see Checklist-Item 5).

**References**

1. Lin, F. *et al.* Near-unity emission in zero-dimensional Sb(III)-based halides intervened by hydrogen bonds towards efficient solid-state lighting technology. *J. Alloys Compd.* **976**, 173054 (2024).
2. Zhao, J.-Q. *et al.* Stepwise Crystalline Structural Transformation in 0D Hybrid Antimony Halides with Triplet Turn-on and Color-Adjustable Luminescence Switching. *Research* **6**, 0094 (2023).
3. Luo, J. J. *et al.* Efficient and stable emission of warm-white light from lead-free halide double perovskites. *Nature* **563**, 541-545 (2018).
4. Song, K. S. & Williams, R. T. Self-Trapped Excitons. 2nd edn. (Berlin: Springer, 1996). DOI: 10.1007/978-3-642-85236-7.
5. Wang, S. *et al.* Tunable Anisotropic Extrinsic Self-Trapped Exciton Emission in Van Der Waals Layered In_4/3_P_2_S_6_. *Adv. Funct. Mater.* **34**, 2312143 (2024).
6. Li, Y. *et al.* Tuning Electron-Phonon Coupling Interaction for the Efficient Wide Blue Emission of Pb^2+^-Doped Cs_2_InCl_5_·H_2_O. *Adv. Opt. Mater.* **12**, 2400184 (2024).
7. Liu, Y. *et al.* Achieving Color-Tunable Long Persistent Luminescence in Cs_2_CdCl_4_ Ruddlesden-Popper Phase Perovskites. *Angew. Chem. Int. Ed.* **62**, e202308420 (2023).
8. Singh, A. & Mitzi, D. B. Emergence of melt and glass states of halide perovskite semiconductors. *Nat. Rev. Mater.* **10**, 211-227 (2025).
9. Friedrich, H. & Trost, J. Nonintegral Maslov indices. *Phys. Rev. A* **54**, 1136-1145 (1996).
10. Zhao, H. G. *et al.* Gram-scale synthesis of carbon quantum dots with a large Stokes shift for the fabrication of eco-friendly and high-efficiency luminescent solar concentrators. *Energy Environ. Sci.* **14**, 396-406 (2021).
11. Meinardi, F. *et al.* Highly efficient luminescent solar concentrators based on earth-abundant indirect-bandgap silicon quantum dots. *Nat. Photonics* **11**, 177-185 (2017).
12. Wu, K. F., Li, H. B. & Klimov, V. I. Tandem luminescent solar concentrators based on engineered quantum dots. *Nat. Photonics* **12**, 105-110 (2018).
13. Wei, M. Y. *et al.* Ultrafast narrowband exciton routing within layered perovskite nanoplatelets enables low-loss luminescent solar concentrators. *Nat. Energy* **4**, 197-205 (2019).
14. Zhao, H. G. *et al.* Zero-Dimensional Perovskite Nanocrystals for Efficient Luminescent Solar Concentrators. *Adv. Funct. Mater.* **29**, 1902262 (2019).
15. Luo, X., Ding, T., Liu, X., Liu, Y. & Wu, K. Quantum-Cutting Luminescent Solar Concentrators Using Ytterbium-Doped Perovskite Nanocrystals. *Nano Lett.* **19**, 338-341 (2018).
16. Li, Z. L. *et al.* Solvent-Solute Coordination Engineering for Efficient Perovskite Luminescent Solar Concentrators. *Joule* **4**, 631-643 (2020).
17. Liu, Y. Q. *et al.* Stable metal-halide perovskites for luminescent solar concentrators of real-device integration. *Nano Energy* **85**, 105960 (2021).
18. Meinardi, F. *et al.* Doped Halide Perovskite Nanocrystals for Reabsorption-Free Luminescent Solar Concentrators. *ACS Energy Lett.* **2**, 2368-2377 (2017).
19. Zhao, H. G. *et al*. Perovskite quantum dots integrated in large-area luminescent solar concentrators. *Nano Energy* **37**, 214-223 (2017).
20. Bagherzadeh-Khajehmarjan, E. *et al*. Bulk luminescent solar concentrators based on organic-inorganic CH_3_NH_3_PbBr_3_ perovskite fluorophores. *Sol. Energy Mater. Sol. Cells* **192**, 44-51 (2019).
21. Meinardi, F. *et al.* Large-area luminescent solar concentrators based on ‘Stokes-shift-engineered’ nanocrystals in a mass-polymerized PMMA matrix. *Nat. Photonics* **8**, 392-399 (2014).
22. Meinardi, F. *et al.* Highly efficient large-area colourless luminescent solar concentrators using heavy-metal-free colloidal quantum dots. *Nat. Nanotechnol.* **10**, 878-885 (2015).
23. Bergren, M. R. *et al.* High-Performance CuInS_2_ Quantum Dot Laminated Glass Luminescent Solar Concentrators for Windows. *ACS Energy Lett.* **3**, 520-525 (2018).
24. Chou, C.-H., Hsu, M.-H. & Chen, F.-C. Flexible luminescent waveguiding photovoltaics exhibiting strong scattering effects from the dye aggregation. *Nano Energy* **15**, 729-736 (2015).
25. Wang, A. F. *et al*. Dendrimer-Encapsulated Halide Perovskite Nanocrystals for Self-Powered White Light-Emitting Glass. *J. Am. Chem. Soc.* **145**, 28156-28165 (2023).
