# Supplementary material for: Recyclable luminescent solar concentrator from lead-free perovskite derivative
Source: Light Sci Appl. 2025 Aug 28;14:297. doi: 10.1038/s41377-025-01973-0 (PMC12394575; doi:10.1038/s41377-025-01973-0)
Supplement: Supplementary file 2 — Supplemantary Checklist [file 41377_2025_1973_MOESM2_ESM.docx]

**Supplementary Checklist for**

**Recyclable luminescent solar concentrator from lead-free perovskite derivative**

Huanxin Yang, Haolin Lu, Xuejiao Wang, Wenda Sun, Yujing Yang, Wei Xiong, Guankui Long, Jialiang Xu, Xiaodan Zhang, Mingjian Yuan, Xiyan Li*

**Item 1.** Have the current density-voltage (*J-V*) characteristics been provided to calculate the power conversion efficiency (*PCE*) of the luminescent solar concentrator-photovoltaic (LSC-PV) systems? The type of the side-mounted PV (i.e., Si, GaAs, CIGS, perovskite or dye-sensitized solar cells) should be clearly addressed, and the corresponding PV performance parameters and spectral response (*EQE_PV_*) of the side-mounted PV cells should also be reported. Does the edge-mounted

PV show any clear hysteresis in the corresponding *J-V* characteristic? If so, stabilized *PCE* near the maximum power point (MPP) should be provided, along with the corresponding *J-V* curves, identifying scan speed and direction. The *J-V* characteristics of an LSC-PV system devoid of emitters (i.e., a blank) under the same testing condition should also be provided.

**Response 1.** Confirmed. All of the parameters and corresponding curves have been provided in this work. No hysteresis has been observed.

**Item 2.** Is the area of the lightguide front surface (*A_LSC_*) used for the photocurrent density and *PCE* calculations? Please provide the dimensions (length, width, and thickness) of the lightguide and the

edge-mounted PV cell. Based on these, the geometric gain (G = *A_LSC_*/*A_Edge_*, where *A_Edge_* is the entire edge area, not only the area mounted with PV cells) should also be provided for reference. Using a square-shaped LSC lightguide with length of at least 5 cm or larger is highly recommended.

**Response 2.** Confirmed. Taking the 5×5×0.5 cm^3^ of G-ETP_2_SbCl_5_ as an example, *A*_LSC_ and *A*_Edge_ are 25 cm^2^ and 10 cm^2^, respectively. Then, G = 25/10 = 2.5.

**Item 3.** For *J-V* measurements, please address the number of lightguide edges mounted with PV cells. Are the unmounted edges taped/painted black or covered with reflectors to block the incident illumination during *J-V* measurements? Any blackened edges should be roughened or applied with index matching gel to the blackened surface to avoid reflections. If reflectors are mounted onto the rest of the edges, no multiplication correction factor should be applied for the *J-V* characteristics. Please indicate whether and which type of index matching material was utilized between the lightguide and the PV cells.

**Response 3.** Confirmed. In this work, the Si solar cells were directly mounted onto the edges without any extra materials. During the *J*-*V* measurements, the unmounted edges were taped black, and a black cardboard was placed under LSCs to prevent reflection.

**Item 4.** For *J-V* measurements, has an opaque mask or aperture with well-defined area been placed directly and closely in front of the LSC lightguide to minimize the impact from any direct incident light on the edge-mounted PV? Has a matte black backdrop been placed behind the tested LSC lightguide to avoid double-pass of light as a baseline measurement?

**Response 4.** Confirmed. An opaque black paper with a well-defined aperture was used during the measurements, and the size of aperture corresponded with the surface size of LSC device. Also, another black cardboard was placed under the LSC device to avoid light reflection.

**Item 5.** Has position-dependent or average external quantum efficiency (*EQE_LSC_(λ)*) data for the LSC-PV system been provided? Has any geometric correction factor been applied to correct the measured *EQE_LSC_* profile? Does the photocurrent density integrated from the average *EQE_LSC_* ($J_{SC}^{Int}$) match the short-circuit current density extracted from the corresponding *J-V* characteristic (*J_SC_*)? The current density discrepancy should not exceed 20%.

**Response 5.** Unfortunately, EQE measurement was not applicable in this work.


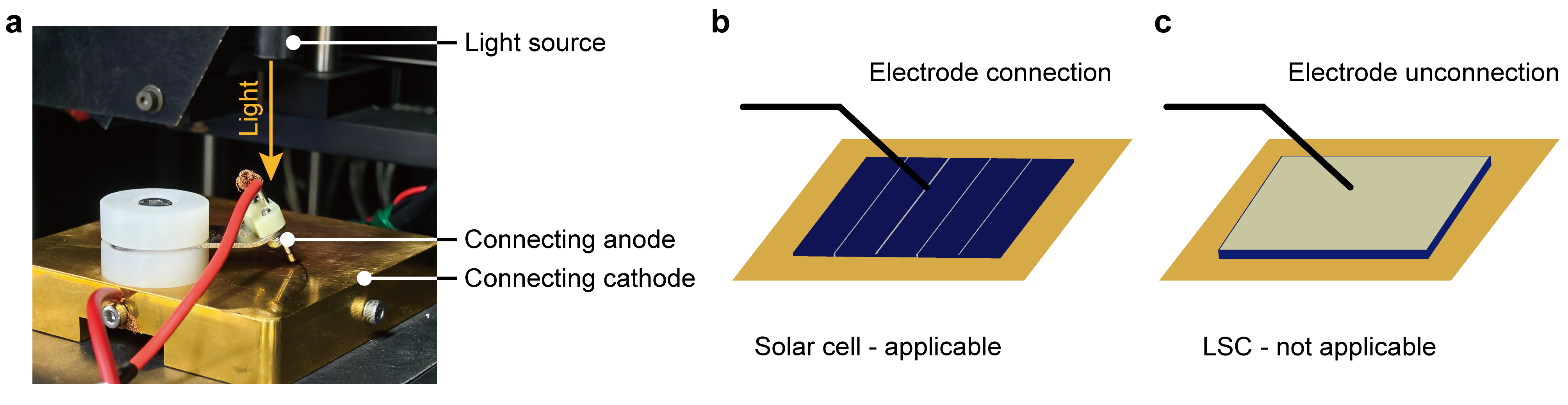


**Fig. C1.** EQE system and measurements for solar cell and LSC device.

It should be noted that for the EQE measurement, most of the research groups measure the EQE values with the set-up shown in Fig. C1a. The electrodes for anode and cathode are fixed and cannot be rotated. For a conventional EQE test for a solar cell, the electrodes could connect the anode and cathode from above and below, respectively (Fig. C1b). However, for a LSC device, the solar cells are coupled at the edges, and the electrodes are distributed sideways instead of up and down, which prevents the electrodes from contacting the terminals of the EQE system, thereby complicating the testing process (Fig. C1c). We believe a specific set-up needs to be further designed for LSC measurement.

**Item 6.** Please state the light source and the reference cell used for the *J-V* characteristic. We recommend providing the intensity and the spectrum of the test light source (AM1.5 G, 1000 W/m^2^ at 25 °C are highly recommended). Inclusion of the illumination beam homogeneity over the testing area is also encouraged. Based on the spectrum of the test light source and the average *EQE_LSC_(λ)* of the LSC_PV_ system, what is the calculated spectral mismatch factor (*M*)?

**Response 6.** Confirmed. The solar simulator equipped with the AM1.5 G light source, of which the power was confirmed as 100 mW cm^-2^. The spectrum of the solar simulator is shown in Fig. C2. EQE measurement was not available in this work.


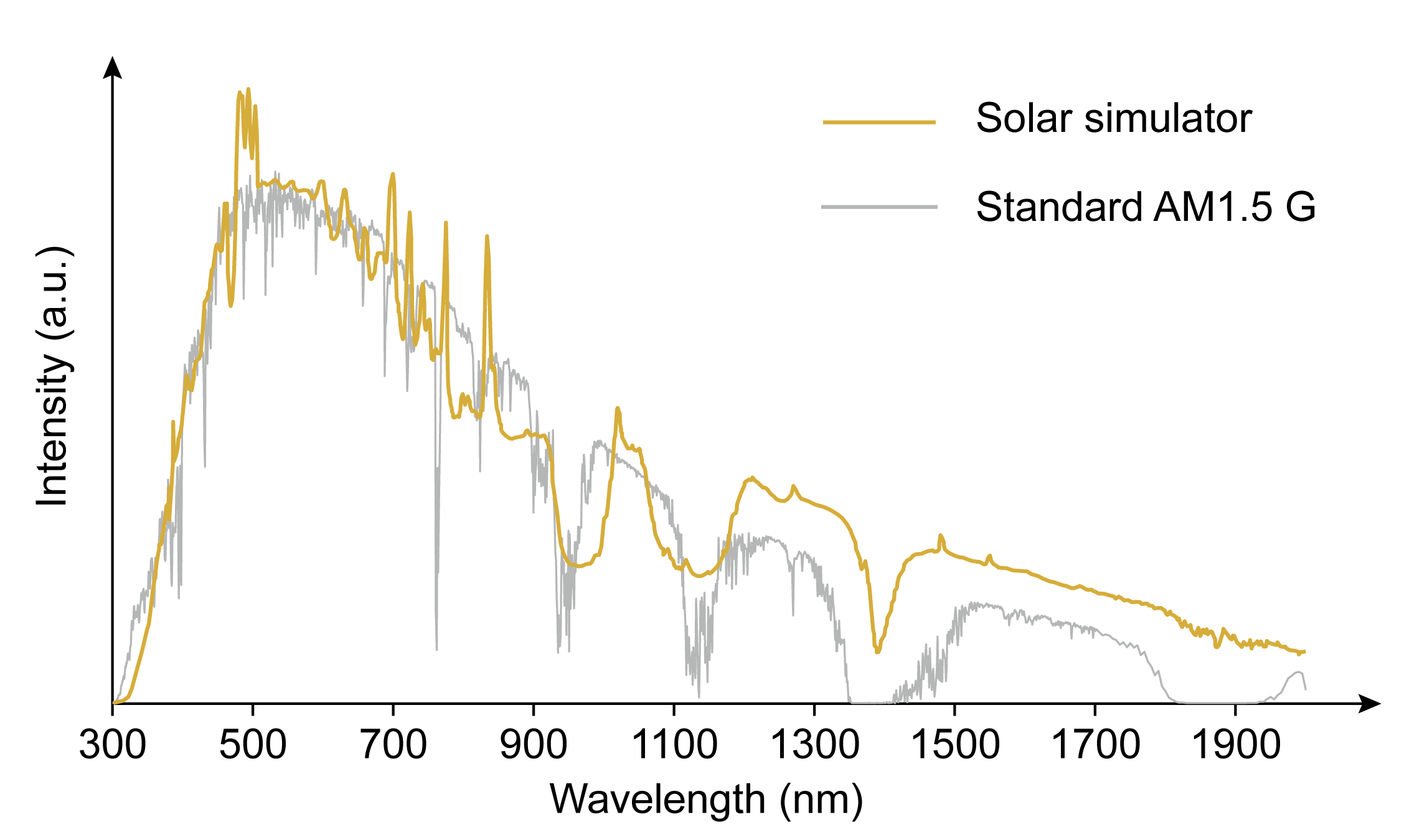


**Fig. C2.** Spectral comparison between solar simulator and standard AM1.5 G.

**Item 7.** Please provide the absolute absorptance spectrum of the entire device (e.g., *A(λ)* = 1 - *T(λ)* - *R(λ)*, where *T(λ)* and *R(λ)* are the transmittance and reflectance spectra of the entire device, respectively), normalized emission spectrum, and the photoluminescence quantum yield (*PLQY*) for the luminophores in the lightguide matrix at the selected concentration. Does the *EQE_LSC_(λ)* peak wavelength match the absorption peak wavelength? Is the peak value of the absolute *EQE_LSC_* limited by the corresponding *PLQY* (i.e., *EQE_LSC_* < *PLQY*)?

**Response 7.** Confirmed. In the manuscript, the absolute absorption spectrum was provided. However, similar as the item 5, EQE measurement was not available in this work.

**Item 8.** Please provide a photon balance check: *T(λ)* + *R(λ)* + *EQE_LSC_(λ)/m* < 1, where *m* accounts for down-converting, multi-exciton generation, up-conversion, quantum cutting, or singlet fission cases for the LSC-PV system, and *EQE_LSC_(λ)* is the maximum position-dependent *EQE_LSC_*. If a double-beam spectrometer is used to measure the transmittance spectrum, please confirm that no blank sample is placed on the reference side.

**Response 8.** Similar to the item 5, the EQE measurement was not available in this work.

**Item 9.** How many LSC-PV devices have been fabricated and tested for the statistical analysis of the photovoltaic performance? Has a stability analysis been performed? If so, please address the test conditions used for this characterization in detail (for example, light source type and intensity, temperature and humidity, contained in inert gas or exposed to ambient air environment, indoors vs. outdoors, under open-circuit, short-circuit, MPP, or stabilized MPP conditions). Data from at least a second measurement 24 h after the initial test is recommended in the same identical conditions, clearly specifying the storing conditions.

**Response 9.** Confirmed. The stability measurements under different conditions were specifically carried out in this work.

**Item 10.** Is transparency an attribute of the LSC-PV system? If so, please provide the average visible transmittance (*AVT*) calculated from the corresponding transmittance (*T(λ)*) of the entire device. If a double-beam spectrometer is used to measure the transmittance spectrum, please confirm that no blank sample is placed on the reference side. Is aesthetic quality from either side an attribute of the LSC-PV system? If so, please provide color rendering index (*CRI*) or CIELAB coordinates (*a*, b**) calculated from the appropriate transmittance and/or reflectance spectrum of the entire device.

**Response 10.** Confirmed. The AVT value for G-ETP_2_SbCl_5_ is ~78.3%, exhibiting great transparent property. The CIE coordinate from transmission spectrum is (0.3685, 0.3889), approximately, shown in Fig. C3.


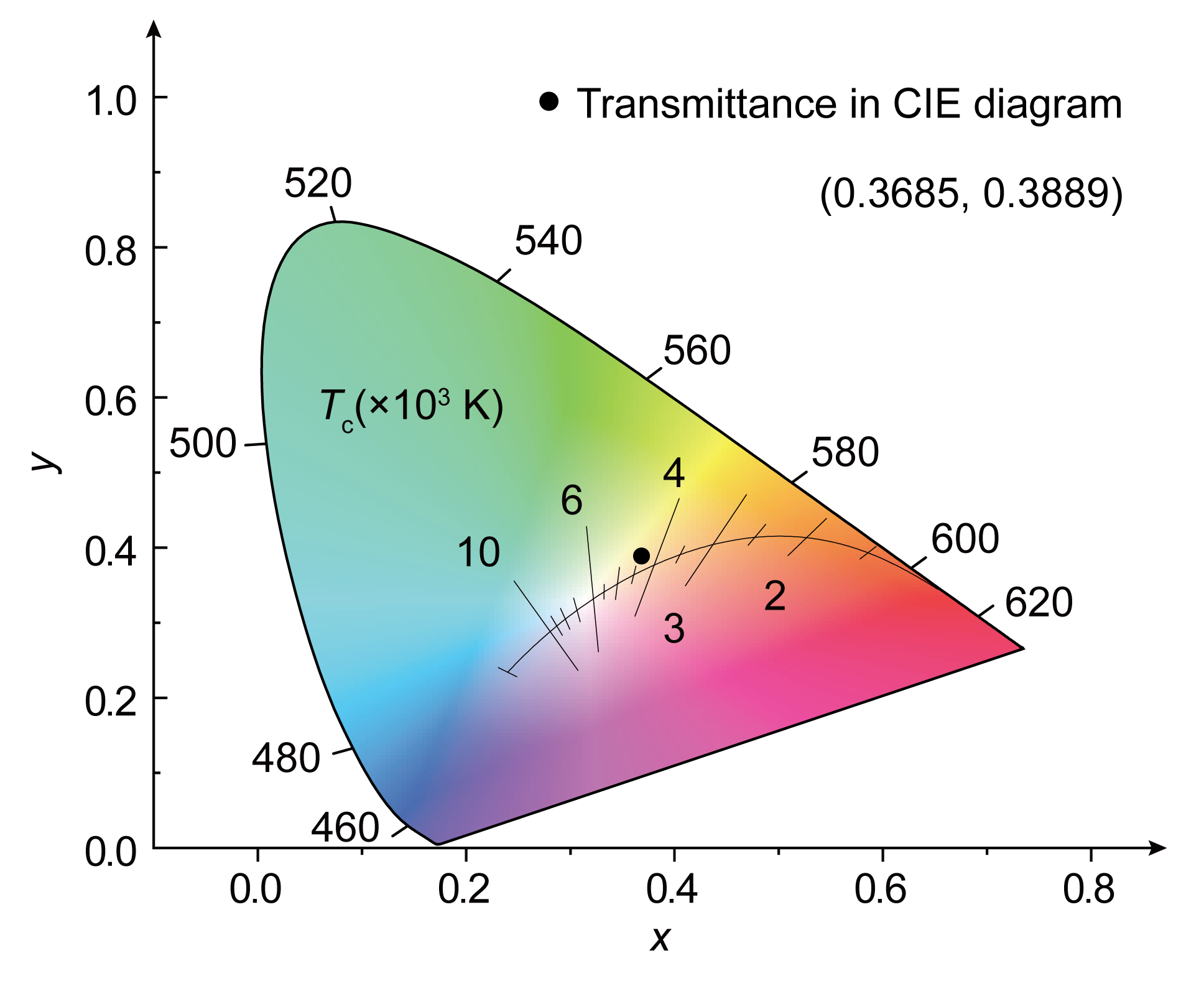


**Fig. C3. CIE diagram for the transmission spectrum.** The black point corresponds to the transmittance spectrum of G-ETP_2_SbCl_5_.
